# A Reassessment of Bergmann's Rule in Modern Humans

**DOI:** 10.1371/journal.pone.0072269

**Published:** 2013-08-28

**Authors:** Frederick Foster, Mark Collard

**Affiliations:** Human Evolutionary Studies Program and Department of Archaeology, Simon Fraser University, BC, Canada; University of Delaware, United States of America

## Abstract

It is widely accepted that modern humans conform to Bergmann's rule, which holds that body size in endothermic species will increase as temperature decreases. However, there are reasons to question the reliability of the findings on which this consensus is based. One of these is that the main studies that have reported that modern humans conform to Bergmann's rule have employed samples that contain a disproportionately large number of warm-climate and northern hemisphere groups. With this in mind, we used latitudinally-stratified and hemisphere-specific samples to re-assess the relationship between modern human body size and temperature. We found that when groups from north and south of the equator were analyzed together, Bergmann's rule was supported. However, when groups were separated by hemisphere, Bergmann's rule was only supported in the northern hemisphere. In the course of exploring these results further, we found that the difference between our northern and southern hemisphere subsamples is due to the limited latitudinal and temperature range in the latter subsample. Thus, our study suggests that modern humans do conform to Bergmann's rule but only when there are major differences in latitude and temperature among groups. Specifically, groups must span more than 50 degrees of latitude and/or more than 30°C for it to hold. This finding has important implications for work on regional variation in human body size and its relationship to temperature.

## Introduction

Bergmann's rule is an empirical generalization concerning body size in endothermic species. It holds that within such species body size varies such that individuals occupying colder environments tend to be larger than individuals who live in warmer environments [1]. This pattern is usually explained in relation to heat production and loss [2]–[9]. According to this hypothesis, a large body is advantageous in cold conditions not only because it has more cells and therefore produces more heat than a smaller body, but also because the relationship between volume and surface area is such that a larger body loses less heat per unit volume than a smaller body. As such, natural selection can be expected to act in such a way that members of a species living in cold environments will be larger than conspecifics occupying warmer environments.

Over the last 50 years, many anthropologists have concluded that modern humans are one of the many species that conform to Bergmann's rule [2]–[5], [7]–[12]. Today, this idea is so widely accepted that it is presented as a fact in many anthropology textbooks [13]–[16]. However, there are several reasons to question the reliability of the findings on which this consensus is based. One of the most important of these is that the main studies that have found the correlation between modern human body size and temperature predicted by Bergmann's rule have employed samples that contain a disproportionately large number of warm-climate and northern hemisphere groups [3], [7], [8], [12], [17]. This raises the possibility that the studies' results primarily reflect the relationships between body size and temperature in warm-climate and/or northern hemisphere groups rather than in *Homo sapiens* as a whole. Given how important the notion that modern humans conform to Bergmann's rule is for our understanding of contemporary human variation, there is a pressing need to determine whether this is in fact the case.

Here, we report a study in which we re-tested the hypothesis that modern humans conform to Bergmann's rule while controlling for the aforementioned sample biases. We carried out three sets of analyses in the study. In the first, we replicated the approach employed in the main studies that have found a correlation between modern human body size and temperature and analyzed the entire sample [7], [12], [17]. In the second, we used stratified sampling to examine the relationship between modern human body size and temperature while controlling for the warm-climate bias in our sample. In the third and final set of analyses, we investigated the relationship between modern human body size and temperature separately in the northern and southern hemispheres. The goal of this set of analyses was to shed light on the impact of the northern hemisphere sample bias in our sample.

## Materials and Methods

**Figure 1:**
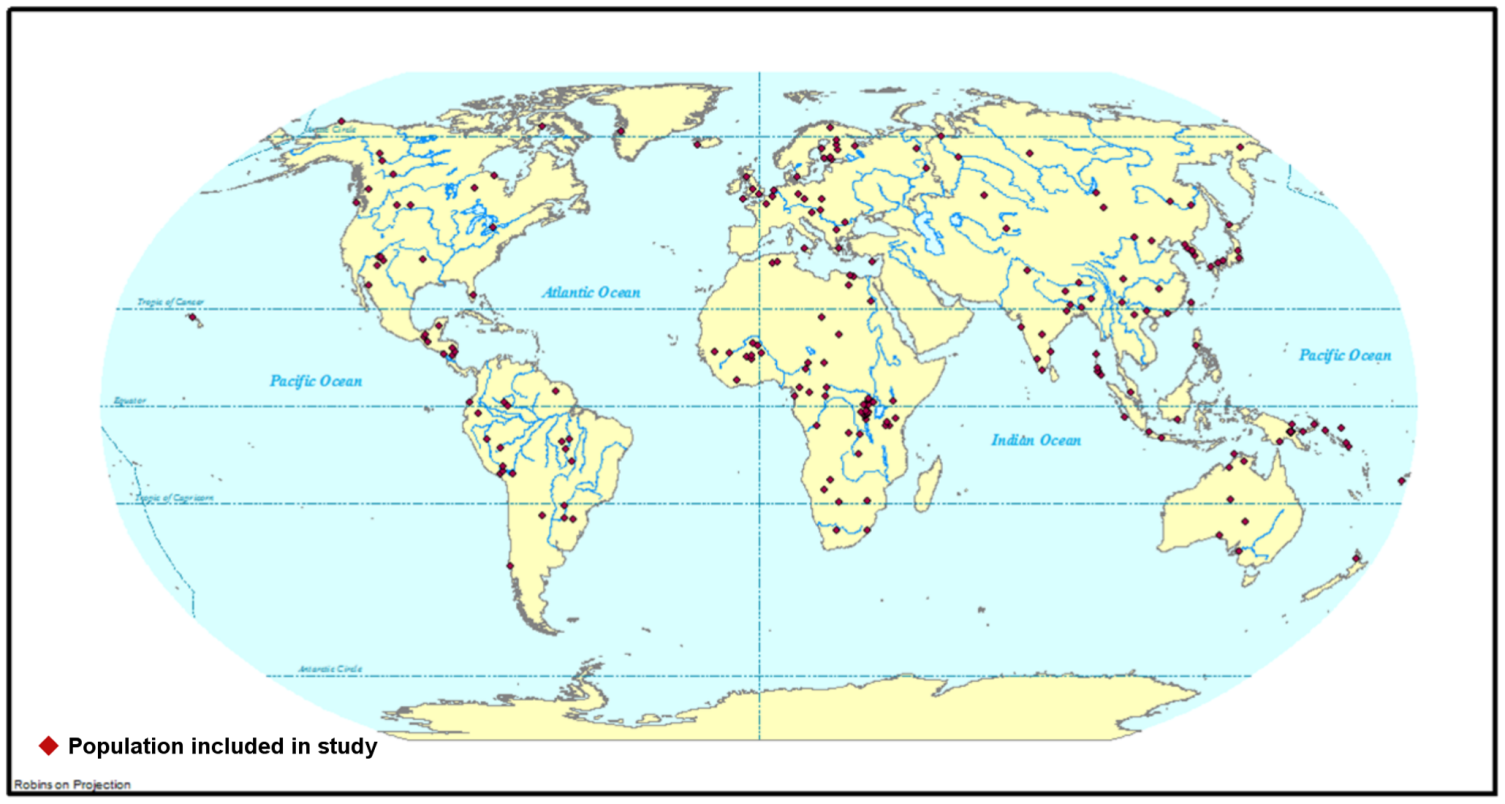
Geographic locations of groups included in the present study.

The sample comprised 263 groups. Details of the groups are given in Supplementary Table 1. To eliminate the effects of inter-group variation in sexual dimorphism, only males were included in the sample. An effort was made to reduce the effects of recent migration by including only groups believed to have resided in their present location since 1492. A group had to be represented by at least ten individuals in order to be included in the sample. As with the samples used in previous global-scale analyses of Bergmann's rule in modern humans [7], [12], [17], a disproportionately large number of groups are from warm climates and the northern hemisphere. This can be seen in Figure 1, which shows the approximate locations of the groups in the sample. A few populations are represented more than once in the dataset. This is because an author listed more than one body mass value for a population without explanation, or because different authors provided different body masses for a population. In these cases, we assumed that the different body mass values pertain to different groups of the same population. We elected not to choose between the body mass values and to simply avoid the duplicates in the creation of the stratified subsamples.

**Table 1:** Results of regression analyses using the unstratified global sample (n = 263). See Materials and Methods for details of abbreviations.

		Body mass(kg)	BMI(kg/m^2^)	SA/BM(cm^2^/kg)	PI(kg/m^3^)
**Absolute latitude**	**r^2^**	0.331	0.196	0.264	0.067
	**p-value**	0.000	0.000	0.000	0.000
**Mean annual temperature (°C)**	**r^2^**	0.262	0.191	0.234	0.086
	**p-value**	0.000	0.000	0.000	0.000

Currently, there is no consensus concerning the variables that should be used to examine the relationship between modern human body size and temperature. Bergmann's rule was initially formulated in relation to body mass [1] and this variable has been used by a number of researchers [7], [12], [17]. But several other variables have also been used to assess the extent to which modern humans conform to Bergmann's rule, including height, the Body Mass Index (BMI; body mass divided by the square of height), the ratio of surface area to body mass (SA/BM), and the Ponderal Index (PI; body mass divided by the cube of height) [3], [7], [10], [18], [19]. There is also ambiguity regarding which variable should be used to represent temperature. Latitude has been employed in some studies [7], [20]; mean annual temperature in others [8], [9], [10], [12], [17], [21]; and both latitude and mean annual temperature in still others [22], [23]. Given these uncertainties, we opted to use multiple body size variables and two temperature variables.

We used body mass, BMI, SA/BM, and PI as our body size varaibles in order to make our study comparable with previous work on Bergmann's rule [7], [8], [9], [10], [12], [17]. We did not include height as a body size variable in our analyses because previous studies have found that variation in height is not associated with latitude and temperature when other body size variables are controlled for [7], [17].

Including BMI, SA/BM, and PI as thermoregulatory variables necessitated the collection of values for height as well as for body mass. The majority of the body mass and height data were taken from Roberts [17] and Eveleth and Tanner [24], [25]. Data for additional groups were obtained via a literature review. Surface area was estimated using Dubois and Dubois' [26] method. In this method, surface area is derived as follows: surface area (cm^2^)  = 0.007184*H^0.725^*BM^0.425^, where H is height (cm) and BM is body mass (kg). While the Dubois and Dubois [26] method of estimating surface area is nearly 100 years old, recent work suggests that it remains useful for living humans [27].

The temperature variables we utilized are latitude and mean annual temperature. Mean annual temperature for a given group was obtained from the source of the anthropometric data or from the Climate Research Unit's Global Climate Database [28]. When the latitude for a group was not included in the source of the anthropometric data, the group's latitude was determined using additional literature or online resources. When only a general geographic area was described, latitude was based on the group's largest population center or the approximate geographic center of the group's territory.

We carried out three sets of analyses. In the first, we replicated the approach employed in the main studies that have found a correlation between human body size and temperature and analyzed the entire sample [7], [12], [17]. We began by regressing each of the anthropometric variables on latitude. We then regressed each of the anthropometric variables on mean annual temperature.

In the second set of analyses, we used stratified sampling to control for the warm climate bias in the sample. In these analyses, stratification was accomplished by dividing latitude into bands of five degrees and randomly selecting groups from each band, such that as far as possible the resulting stratified sample included eight groups for every five degree band of absolute latitude. Between 30° S and 30° N each five degree band of latitude was equally represented by four groups. Due to a shortage of data from the latitudinal bands between 30° S and 40° S meeting the criteria for inclusion in our dataset, these bands were represented by fewer than four groups. The 30–35° S band was represented by one group, and the 35–40° S band by three groups. To compensate for this, seven groups were randomly selected from the 30–35° N band, and five groups were randomly selected from the 35–40° N band. This ensured that each band of absolute latitude between 0 and 40° was represented by eight groups. Next, eight groups were randomly selected for every five degree band of latitude above 40° N to compensate for the paucity of modern human occupation below 40° S. The only band of absolute latitude with fewer than eight groups was the northernmost, 70–75° N band, which is represented by only one group. Subsequently, we regressed each of the anthropometric variables on latitude and mean annual temperature. This procedure was repeated nine times to counter the negative impacts of reduced sample size.

In the third set of analyses, we controlled for potential biases related to temperature and hemispheric location. As in the previous set of analyses, The effects of the warm climate bias were controlled with stratified sampling. The effects of the northern hemisphere bias were controlled by analysing groups from north and south of the equator separately. For each subsample, four groups were randomly selected for each band of five degrees of latitude. As noted in relation to the previous set of analyses, the 30–35° S and 70–75° N bands of latitude were represented by only one group each, while the 35–40° S band was represented by just three groups. Because the third set of analyses considers the northern and southern hemispheres independently, the groups from these bands were not supplemented by adding additional groups from the opposing hemisphere as was done in the previous analyses. Body mass, BMI, SA/BM, and PI were regressed on latitude and mean annual temperature for the stratified sample. As in the previous set of analyses, the sampling process and analyses were repeated nine times.

Both linear and quadratic regression lines were fitted to the data in each set of analyses because there is reason to think some human traits may exhibit a nonlinear relationship with environmental factors [29]. Quadratic and linear curves were compared using the extra sum-of-squares method. In this methiod, an F-ratio and p-value are calculated and used to evaluate whether the increase in goodness of fit from linear to quadratic is significant when the requirement of an additional parameter is taken into account [30]. All the analyses were carried out in PASW version 18. The p-value was adjusted for multiple unplanned tests using the method outlined by Benjamini and Yekutieli [31]. We employed this method rather than the more commonly used Bonferroni correction because it has been found to be markedly less prone to Type II errors than Bonferroni correction [32].

## Results

A quadratic fit did not significantly improve the characterization of any relationship in any of the analyses. Consequently, we only report the results of the linear regression analyses.

**Figure 2:**
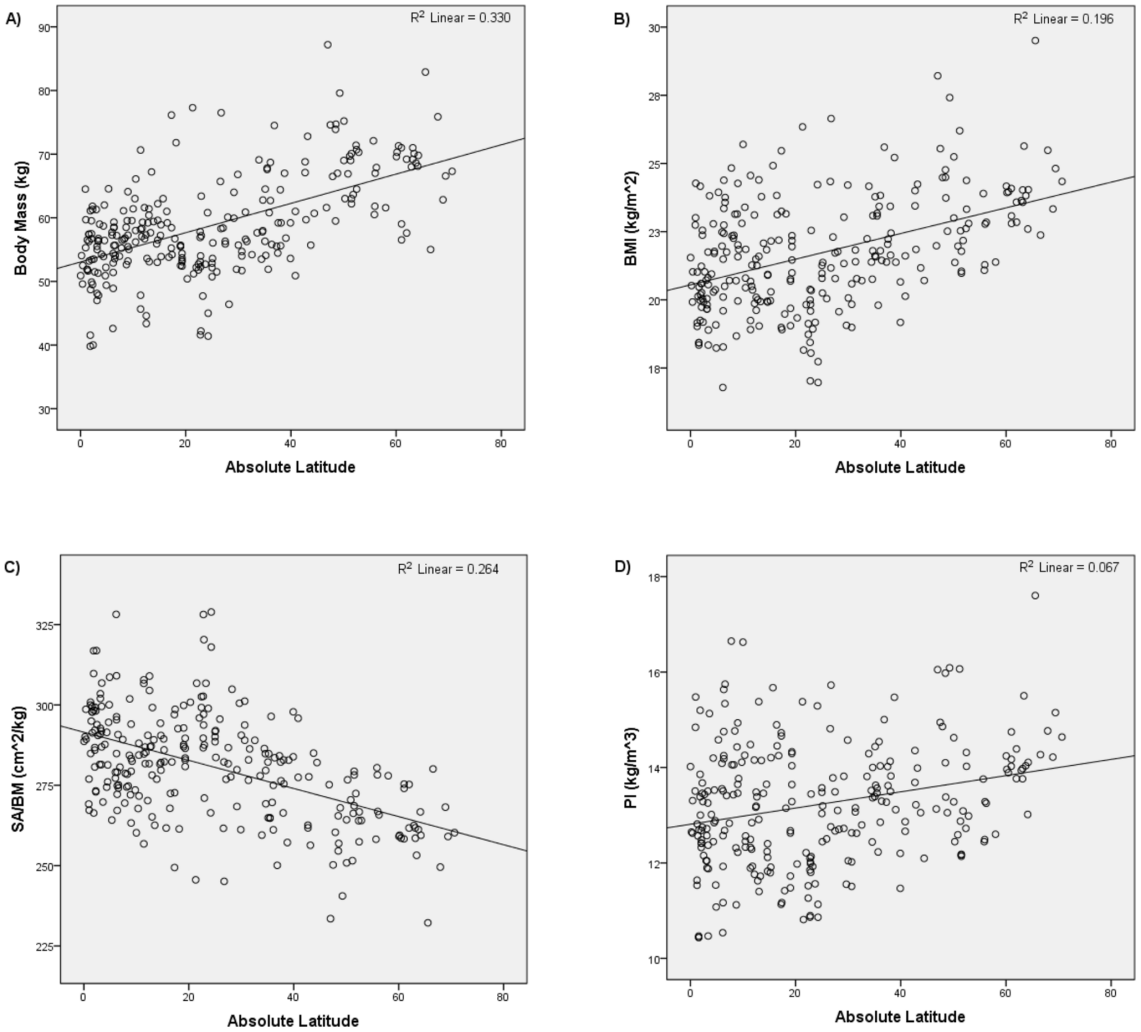
Absolute latitude versus i) body mass, ii) BMI, iii) SA/BM, and iv) PI for global unstratified sample. See Materials and Methods for details of abbreviations.

**Figure 3:**
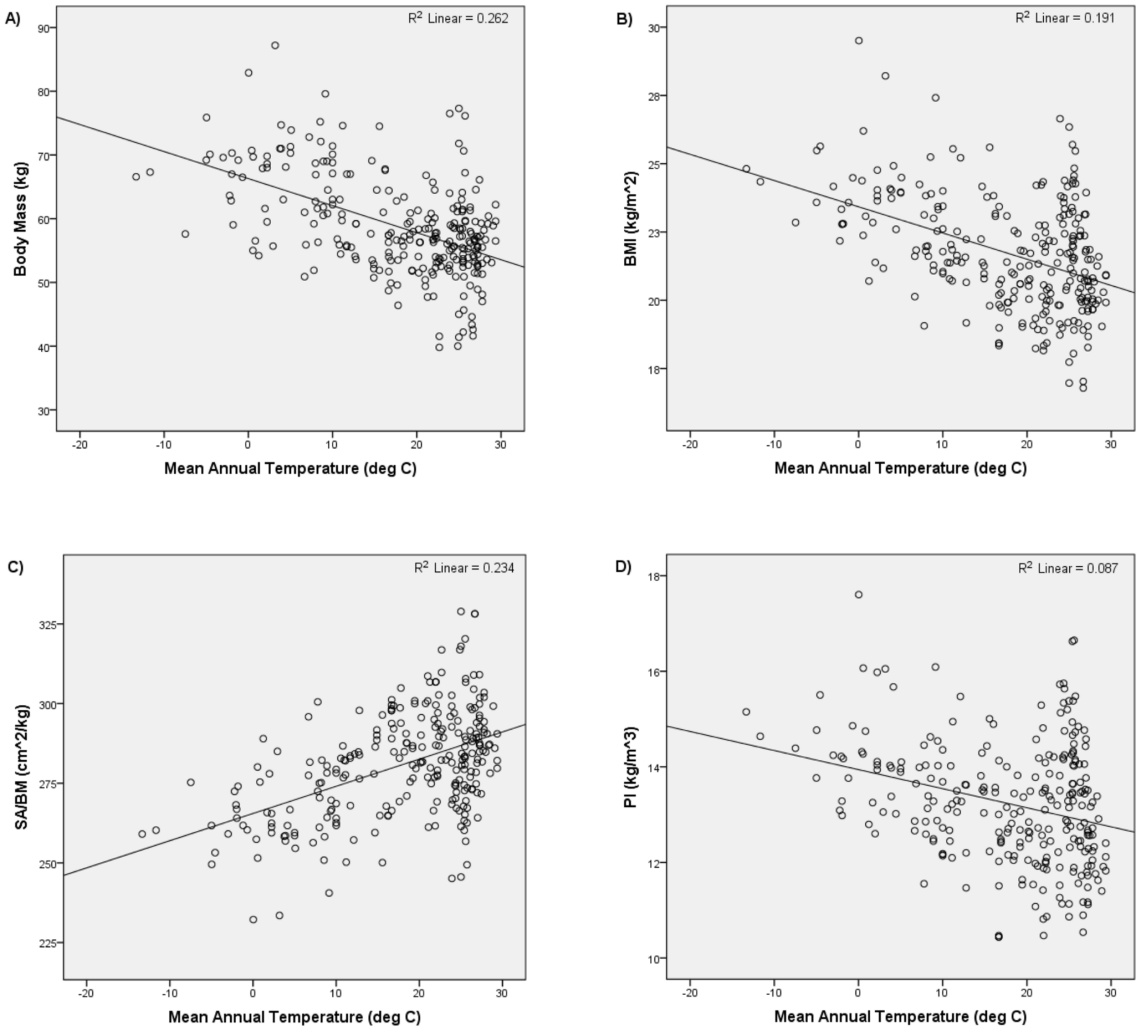
Mean annual temperature versus i) body mass, ii) BMI, iii) SA/BM, and iv) PI for global unstratified sample.

Table 1 and Figures 2 and 3 summarize the results of the first set of analyses. Consistent with Bergmann's rule, body mass, BMI, and PI were all positively and significantly correlated with absolute latitude and negatively and significantly correlated with mean annual temperature. Also consistent with Bergmann's rule, SA/BM was negatively and significantly correlated with absolute latitude and positively and significantly correlated with mean annual temperature. Thus, the analyses that used the unstratified global sample supported the hypothesis that humans conform to Bergmann's rule.

**Figure 4:**
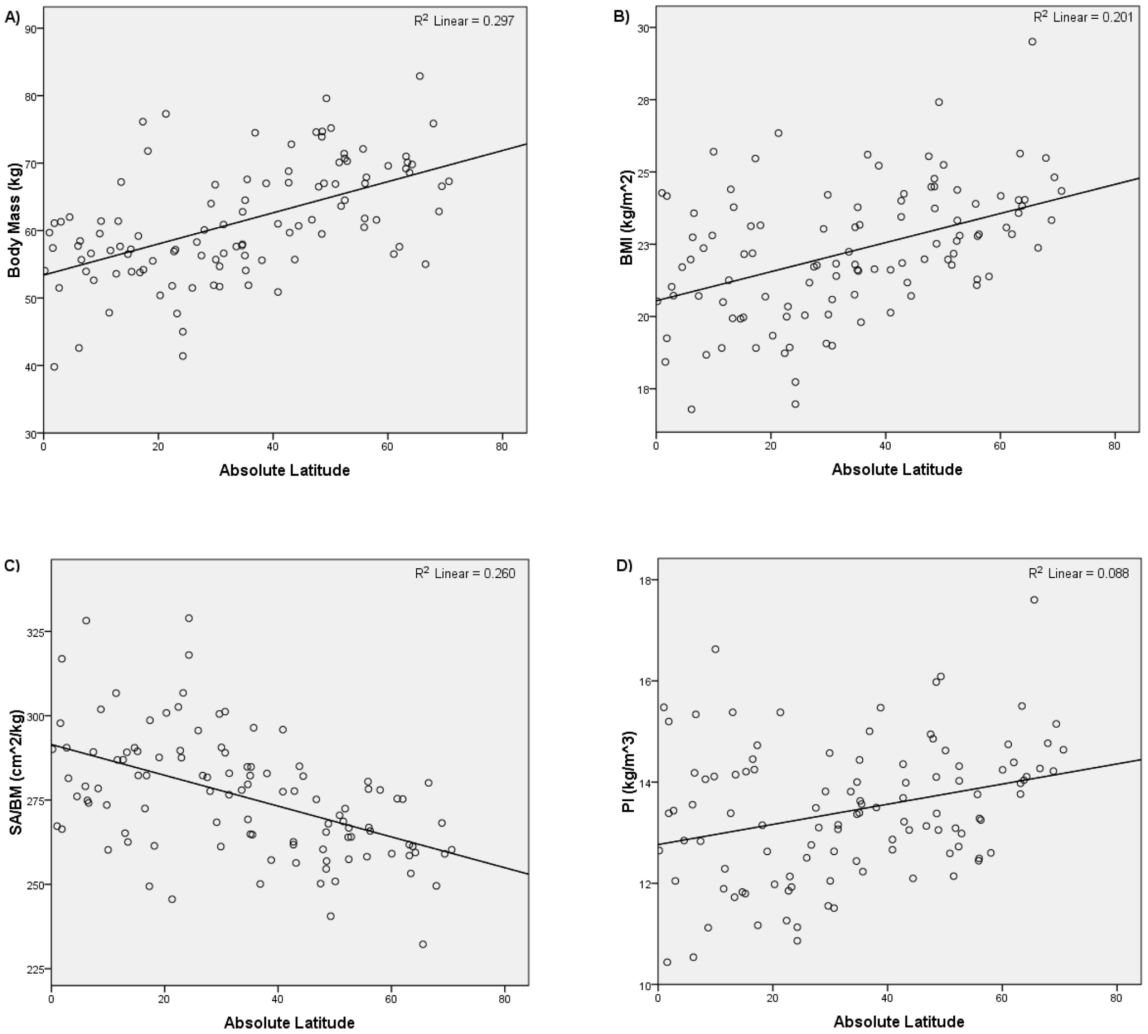
Absolute latitude versus i) body mass, ii) BMI, iii) SA/BM, and iv) PI for one of the stratified global subsamples.

**Figure 5:**
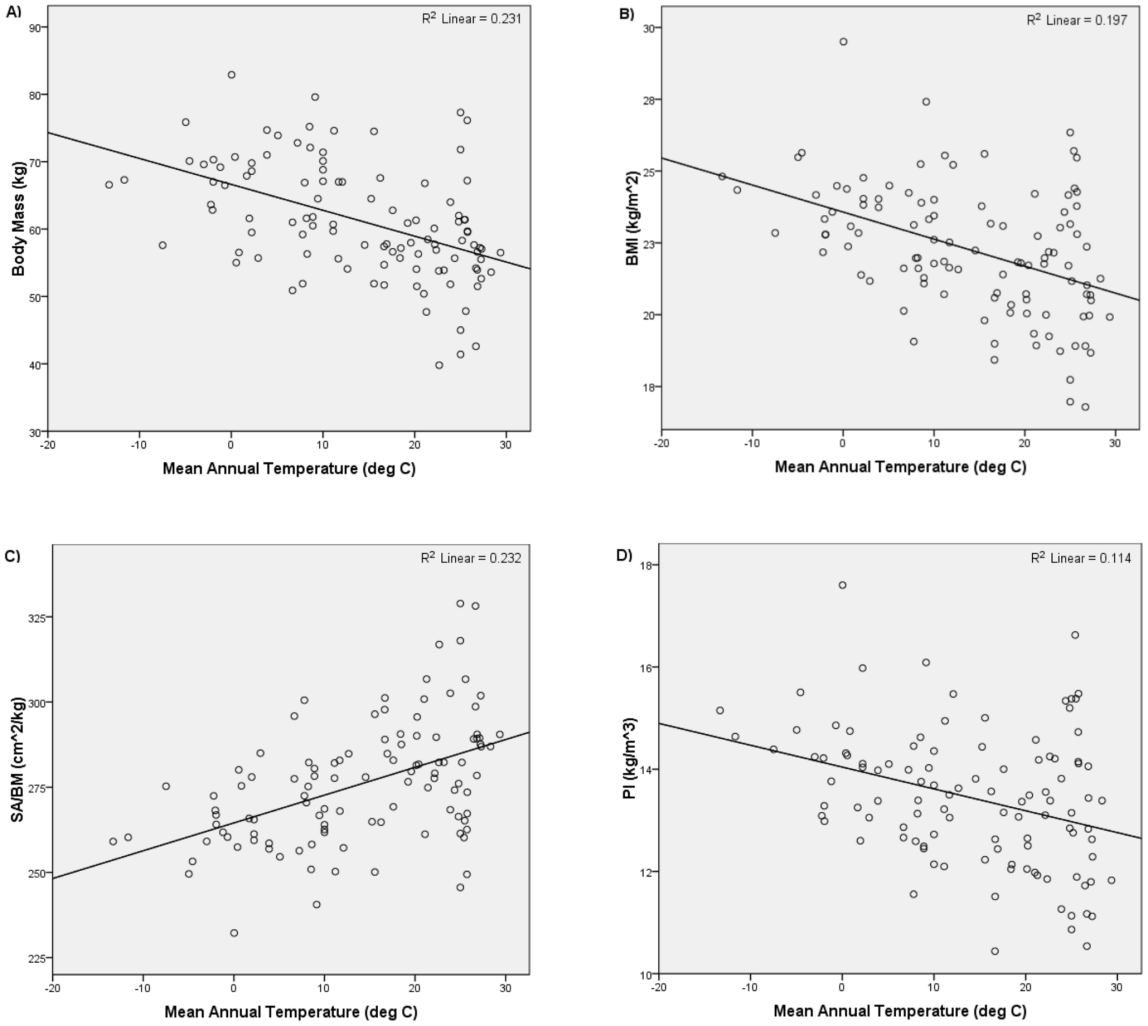
Mean annual temperature versus i) body mass, ii) BMI, iii) SA/BM, and iv) PI for one of the stratified global subsamples.

Table 2 summarizes the results of the second set of analyses. Figures 4 and 5 show the relationships yielded by one of the stratified global subsamples; the other subsamples yielded similar plots. Consistent with Bergmann's rule, the regressions of body mass, BMI, and PI on latitude all returned significant and positive relationships, and the regressions of body mass, BMI, and PI on mean annual temperature all returned significant and negative relationships. The regressions of SA/BM on absolute latitude and mean annual temperature were also consistent with Bergmann's rule. All the regressions of SA/BM on latitude returned a significant and negative relationship, while all the regressions of SA/BM on mean annual temperature returned a significant and positive relationship. Thus, the analyses of the stratified global subsamples also supported the hypothesis that modern humans conform to Bergmann's rule.

**Table 2:** Results of regression analyses using stratified global subsamples (n = 108). All relationships are significant at the Benjamini-Yekutieli adjusted p-value.

		Absolute latitude				Mean annual temperature (°C)			
Subsample		Body mass(kg)	BMI(kg/m^2^)	SA/BM(cm^2^/kg)	PI(kg/m^3^)	Body mass(kg)	BMI(kg/m^2^)	SA/BM(cm^2^/kg)	PI(kg/m^3^)
**1**	**r^2^**	0.306	0.246	0.291	0.146	0.251	0.246	0.272	0.178
	**p-value**	0.000	0.000	0.000	0.000	0.000	0.000	0.000	0.000
**2**	**r^2^**	0.339	0.189	0.282	0.066	0.228	0.187	0.228	0.109
	**p-value**	0.000	0.000	0.000	0.007	0.000	0.000	0.000	0.000
**3**	**r^2^**	0.297	0.201	0.260	0.088	0.231	0.197	0.232	0.114
	**p-value**	0.000	0.000	0.000	0.002	0.000	0.000	0.000	0.000
**4**	**r^2^**	0.302	0.204	0.271	0.098	0.221	0.195	0.230	0.124
	**p-value**	0.000	0.000	0.000	0.001	0.000	0.000	0.000	0.000
**5**	**r^2^**	0.396	0.225	0.327	0.077	0.334	0.243	0.312	0.119
	**p-value**	0.000	0.000	0.000	0.004	0.000	0.000	0.000	0.000
**6**	**r^2^**	0.341	0.189	0.275	0.062	0.235	0.172	0.218	0.084
	**p-value**	0.000	0.000	0.000	0.009	0.000	0.000	0.000	0.002
**7**	**r^2^**	0.309	0.235	0.287	0.125	0.269	0.262	0.287	0.177
	**p-value**	0.000	0.000	0.000	0.000	0.000	0.000	0.000	0.000
**8**	**r^2^**	0.411	0.220	0.326	0.070	0.327	0.232	0.294	0.111
	**p-value**	0.000	0.000	0.000	0.006	0.000	0.000	0.000	0.000
**9**	**r^2^**	0.299	0.211	0.268	0.097	0.219	0.194	0.221	0.116
	**p-value**	0.000	0.000	0.000	0.001	0.000	0.000	0.000	0.001
**10**	**r^2^**	0.312	0.198	0.270	0.072	0.254	0.209	0.251	0.112
	**p-value**	0.000	0.000	0.000	0.005	0.000	0.000	0.000	0.003

**Figure 6:**
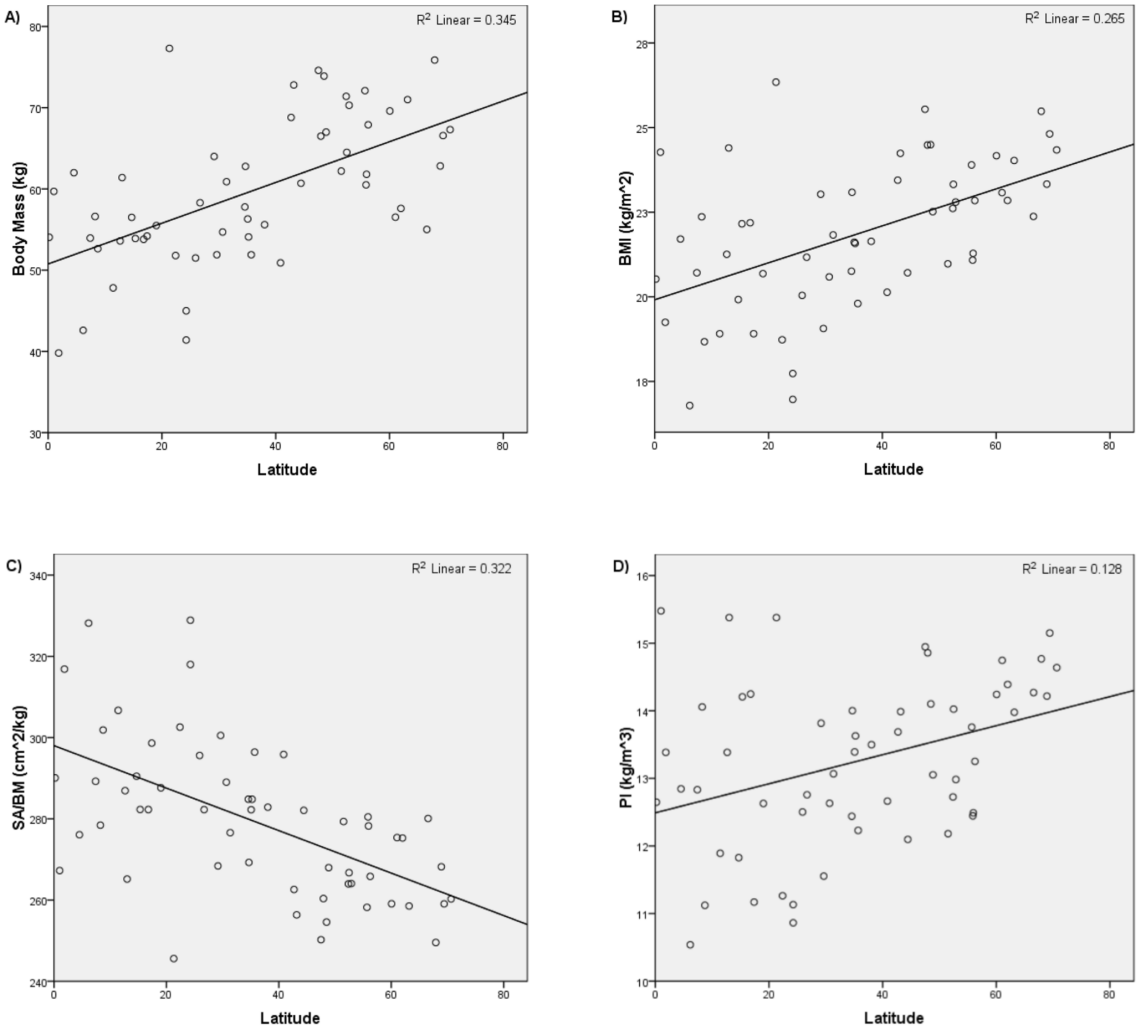
Latitude versus i) body mass, ii) BMI, iii) SA/BM, and iv) PI for one of the stratified northern hemisphere subsamples.

**Figure 7:**
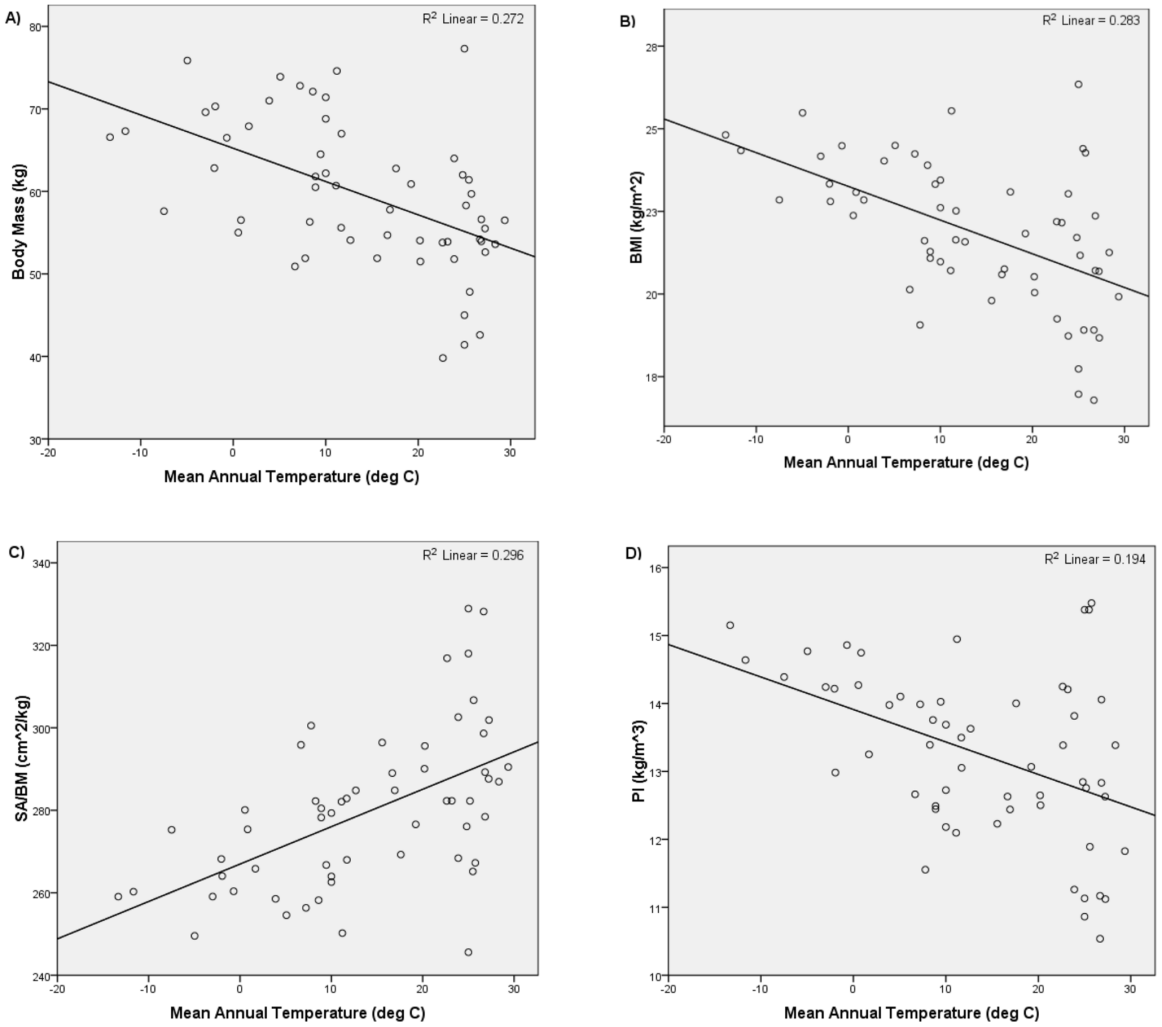
Mean annual temperature versus i) body mass, ii) BMI, iii) SA/BM, and iv) PI for one of the stratified northern hemisphere subsamples.

**Figure 8:**
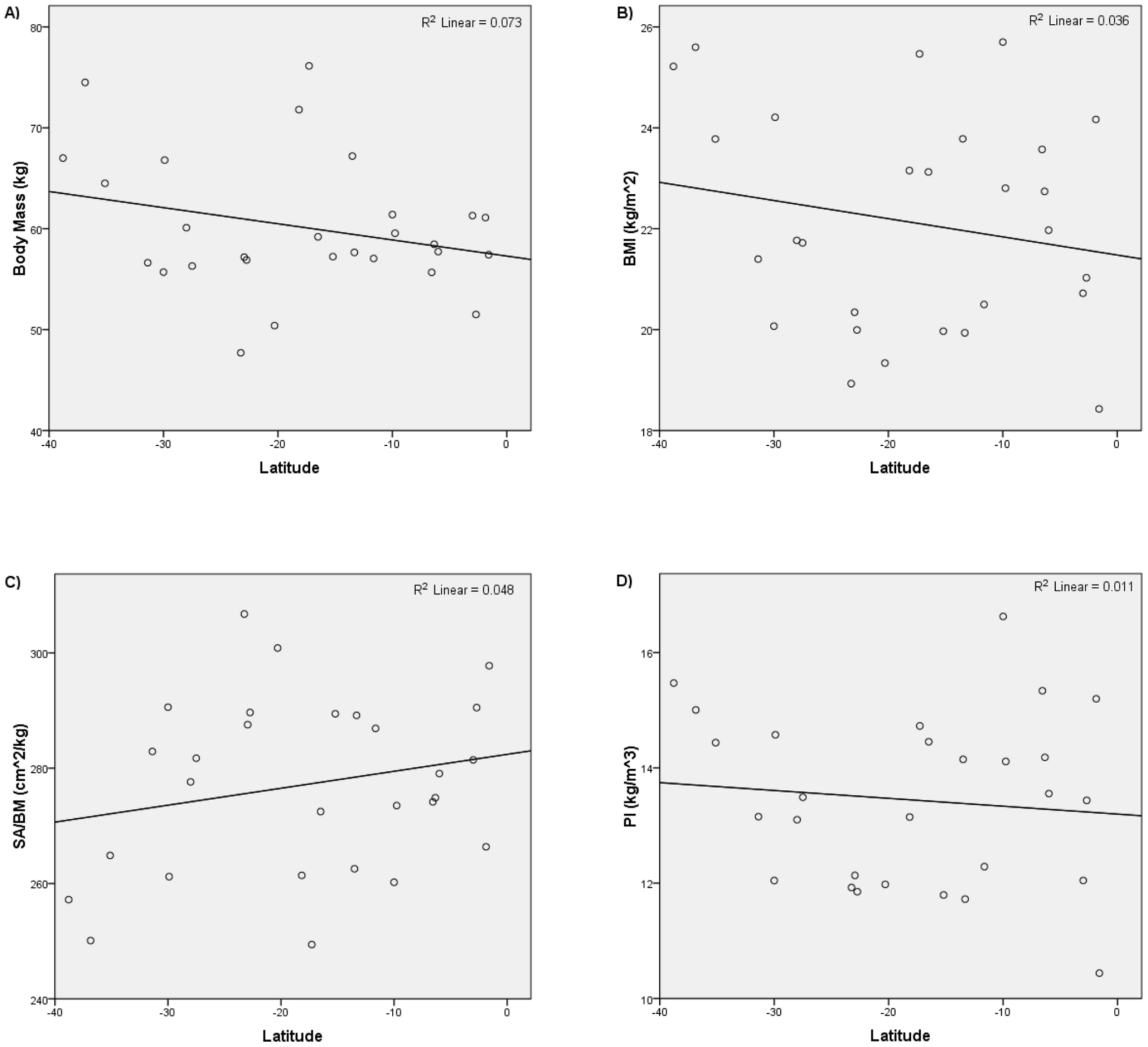
Latitude versus i) body mass, ii) BMI, iii) SA/BM, and iv) PI for one of the stratified southern hemisphere subsamples.

**Figure 9:**
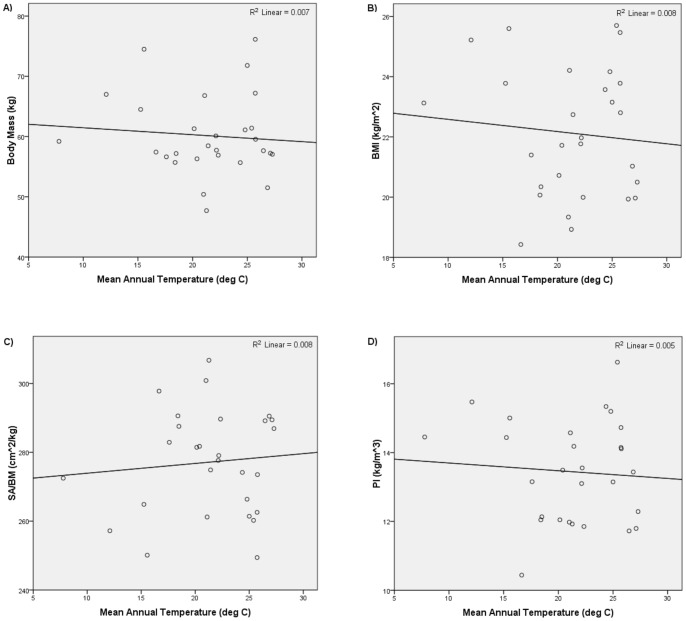
Mean annual temperature versus i) body mass, ii) BMI, iii) SA/BM, and iv) PI for one of the stratified southern hemisphere subsamples.

The results of the third set of analyses are summarized in Tables 3 and 4 and Figures 6–9. Figures 6 and 7 show the relationships yielded by one of the stratified northern subsamples; Figures 8 and 9 show the relationships yielded by one of the stratified southern subsamples. The other stratified northern and southern subsamples yielded similar plots.

**Table 3:** Results of regression analyses using stratified northern hemisphere subsamples (n = 57). All relationships are significant at the Benjamini-Yekutieli adjusted p-value.

		Latitude				Mean annual temperature (°C)			
Subsample		Body mass(kg)	BMI(kg/m^2^)	SA/BM(cm^2^/kg)	PI(kg/m^3^)	Body mass(kg)	BMI(kg/m^2^)	SA/BM(cm^2^/kg)	PI(kg/m^3^)
**1**	**r^2^**	0.405	0.355	0.391	0.231	0.345	0.365	0.382	0.284
	**p-value**	0.000	0.000	0.000	0.000	0.000	0.000	0.000	0.000
**2**	**r^2^**	0.328	0.331	0.372	0.276	0.231	0.228	0.282	0.251
	**p-value**	0.000	0.000	0.000	0.000	0.000	0.000	0.000	0.000
**3**	**r^2^**	0.345	0.265	0.322	0.128	0.272	0.283	0.296	0.194
	**p-value**	0.000	0.000	0.000	0.000	0.000	0.000	0.000	0.001
**4**	**r^2^**	0.406	0.277	0.376	0.136	0.347	0.287	0.348	0.176
	**p-value**	0.000	0.000	0.000	0.005	0.000	0.000	0.000	0.001
**5**	**r^2^**	0.476	0.390	0.464	0.220	0.379	0.345	0.391	0.220
	**p-value**	0.000	0.000	0.000	0.000	0.000	0.000	0.000	0.000
**6**	**r^2^**	0.338	0.235	0.306	0.113	0.292	0.271	0.305	0.178
	**p-value**	0.000	0.000	0.000	0.011	0.000	0.000	0.000	0.001
**7**	**r^2^**	0.321	0.302	0.336	0.203	0.349	0.390	0.405	0.302
	**p-value**	0.000	0.000	0.000	0.000	0.000	0.000	0.000	0.000
**8**	**r^2^**	0.460	0.272	0.374	0.090	0.357	0.290	0.336	0.149
	**p-value**	0.000	0.000	0.000	0.023	0.000	0.000	0.000	0.003
**9**	**r^2^**	0.389	0.293	0.357	0.151	0.339	0.324	0.343	0.219
	**p-value**	0.000	0.000	0.000	0.003	0.000	0.000	0.000	0.001
**10**	**r^2^**	0.366	0.332	0.385	0.002	0.302	0.354	0.359	0.236
	**p-value**	0.000	0.000	0.000	0.002	0.000	0.000	0.000	0.001

**Table 4:** Results of regression analyses using stratified southern hemisphere subsamples (n = 28). Significant relationships at the Benjamini-Yekutieli adjusted p-value are identified in bold.

		Latitude				Mean annual temperature (°C)			
Subsample		Body mass(kg)	BMI(kg/m^2^)	SA/BM(cm^2^/kg)	PI(kg/m^3^)	Body mass(kg)	BMI(kg/m^2^)	SA/BM(cm^2^/kg)	PI(kg/m^3^)
**1**	**r^2^**	0.211	0.171	0.211	0.101	0.001	0.018	0.005	0.038
	**p-value**	0.014	0.029	0.014	0.100	0.865	0.501	0.733	0.322
**2**	**r^2^**	0.263	0.055	0.122	0.002	0.006	0.022	0.013	0.024
	**p-value**	**0.005**	0.229	0.069	0.843	0.696	0.448	0.571	0.426
**3**	**r^2^**	0.073	0.036	0.048	0.011	0.007	0.008	0.008	0.005
	**p-value**	0.166	0.334	0.262	0.597	0.674	0.644	0.645	0.710
**4**	**r^2^**	0.116	0.136	0.128	0.103	0.007	0.004	0.000	0.021
	**p-value**	0..076	0..053	0.062	0.096	0.672	0.736	0.975	0.465
**5**	**r^2^**	0.214	0.098	0.141	0.041	0.113	0.102	0.110	0.073
	**p-value**	0.013	0.104	0.049	0.298	0.080	0.098	0.084	0.165
**6**	**r^2^**	0.262	0.141	0.214	0.039	0.000	0.002	0.001	0.002
	**p-value**	**0.005**	0.049	0.013	0.316	0.921	0.803	0.863	0.826
**7**	**r^2^**	0.193	0.156	0.172	0.096	0.080	0.100	0.090	0.080
	**p-value**	0.019	0.038	0.028	0.109	0.145	0.102	0.122	0.144
**8**	**r^2^**	0.284	0.225	0.256	0.149	0.037	0.094	0.071	0.108
	**p-value**	**0.004**	0.011	**0.006**	0.042	0.324	0.113	0.170	0.088
**9**	**r^2^**	0.131	0.191	0.172	0.169	0.000	0.009	0.004	0.019
	**p-value**	0.058	0.020	0.028	0.030	0.946	0.627	0.744	0.480
**10**	**r^2^**	0.146	0.070	0.097	0.030	0.146	0.070	0.097	0.030
	**p-value**	0.045	0.173	0.107	0.376	0.414	0.389	0.389	0.450

Regressing body mass, BMI, and PI on latitude and mean annual temperature for the northern hemisphere subsample produced results consistent with Bergmann's rule. Significant, positive relationships were returned in all the analyses in which body mass, BMI, and PI were regressed on latitude, while significant, negative relationships were returned in all the analyses in which body mass, BMI, and PI were regressed on mean annual temperature. The results of the analyses in which SA/BM was regressed on latitude and mean annual temperature using the northern hemisphere groups were also consistent with Bergmann's rule. All the analyses in which SA/BM was regressed on latitude returned significant, negative relationships, while all the analyses in which SA/BM was regressed on mean annual temperature yielded significant, positive relationships.

The results of the analyses that focused on southern hemisphere groups were different from the results of those that focused on northern hemisphere groups. Significant, negative relationships were identified in only three of the ten analyses in which body mass was regressed on latitude, and no significant relationships were identified in the analyses in which body mass was regressed on mean annual temperature. For BMI, only one regression on latitude yielded a significant and negative relationship, while none of the regressions on mean annual temperature identified significant relationships. No significant relationships were identified in the analyses in which PI was regressed on either latitude or mean annual temperature. For SA/BM, one regression on latitude identified a significant and positive relationship, while none of the regressions on mean annual temperature identified significant relationships.

Thus, the results of the third set of analyses are inconsistent with the hypothesis that modern humans conform to Bergmann's rule. They suggest that modern human body size is influenced by temperature-driven natural selection in the northern hemisphere but not in the southern hemisphere.

## Discussion

Bergmann's rule was supported when we analyzed the entire sample. It was also supported when we controlled for the warm-climate bias in the sample. However, Bergmann's rule was only partially supported when we controlled for the northern hemisphere bias in the sample. The northern hemisphere groups conformed to Bergmann's rule, but the southern hemisphere groups did not. Thus, our study only partially supported the hypothesis that modern humans conform to Bergmann's rule. This suggests that the hypothesis requires modification.

The nature of the modification depends on the explanation for the difference between the results for the northern hemisphere and southern hemisphere subsamples. There would appear to be three potential explanations for the difference. One is that groups in the southern hemisphere differ from those in the northern hemisphere in such a way that the impact of thermoregulation-related natural selection on body size has been moderated in the former. If, for example, southern hemisphere groups have, on average, migrated into their current location more recently than northern hemisphere groups, there may simply have not been enough time for the predicted relationship between body size and temperature to have evolved. Similarly, there is reason to think that group differences in body mass are partly determined by food availability [12]. Thus, it could be that the southern and northern hemisphere subsamples differ in their access to food in such a way that Bergmann's rule holds for the northern hemisphere subsample but not for the southern hemisphere subsample.

Another potential explanation for the difference between the northern and southern hemisphere subsamples concerns climate. The latitudinal range of the northern hemisphere groups in our sample extends from 0° to 70° N, whereas the latitudinal range of the southern hemisphere groups is 0 to 40° S. This imbalance is likely related to the limited amount of landmass available for modern human occupation in medium to high latitudes in the southern hemisphere compared the equivalent latitudes in the northern hemisphere (Figure 1). Regardless of its cause, the imbalance means that the range of mean annual temperatures associated with the southern groups is more limited than the range of mean annual temperatures associated with the northern groups. The difference between the coldest and warmest mean annual temperatures for the northern hemisphere sample is 43°C, whereas the difference between the coldest and warmest mean annual temperatures for the southern hemisphere is 24°C. Thus, it could be that the range of temperature variation associated with the southern hemisphere groups is insufficient for thermoregulation-related natural selection to have become the dominant influence on variation in modern human body size. This hypothesis was proposed by Hiernaux and Froment [19]. These authors suggested that a limited range of temperature variation likely explained their failure to observe Bergmann's rule in sub-Saharan African groups, which span a mean annual temperature range of only 8°C.

The third potential explanation for the difference between our northern and southern hemisphere groups is also related to the limited amount of habitable land in medium to high latitudes in the southern hemisphere compared to the equivalent latitudes in the northern hemisphere. The ranges of temperature variation in the southern and northern hemispheres differ because the lowest temperature in the northern hemisphere is considerably lower than the lowest temperature in the southern hemisphere. Mean annual temperature for the northern hemisphere subsample ranges from −13 to 29°C, whereas mean annual temperature for the southern hemisphere subsample ranges from 4 to 28°C. As such, it is possible that it is not the limited range of temperature variation that accounts for the failure of the southern hemisphere sample to conform to Bergmann's rule, but rather the absence of mean annual temperatures below 4°C. Stinson [21] outlined this hypothesis. She suggested that the absence of cold climate groups in South America may account for her failure to find a significant association between body mass and latitude or temperature on this continent.

To test between these possibilities, we carried out a set of supplementary analyses in which we divided our northern hemisphere subsample at 40°, which, to reiterate, is the highest latitude represented in our southern hemisphere subsample. The 0–40° N latitudinal range corresponds to a mean annual temperature range of 1 to 29°C, while the >40° N latitudinal range represents a mean annual temperature range of −13 to 12°C. We used random sampling to create a latitudinally-stratified subsample for 0–40° N and one for >40° N. We then regressed body mass, BMI, PI, and SA/BM on latitude and mean annual temperature. This procedure was carried out ten times to ensure repeatability. We reasoned that, if the first hypothesis is correct and some attribute of the southern hemisphere groups prevents them from conforming to Bergmann's rule, then the groups from 0–40° N and the groups from >40° N should both support Bergmann's rule. If the second hypothesis is correct and the issue is insufficient temperature variation in the habitable regions of the southern hemisphere, then neither the groups from 0–40° N, nor the groups from >40° N, should support Bergmann's rule. If the third explanation is correct and it is the absence of temperatures below 4°C that causes the southern hemisphere groups not to conform to Bergmann's rule, the groups from >40° N should support Bergmann's rule while those from 0–40° N should not.

Tables 5 and 6 summarize the results of the supplementary analyses. In the analyses that focused on groups from 0–40° N no significant relationships were identified between the anthropometric variables and latitude, or between the anthropometric variables and mean annual temperature. In the analyses that focused on groups from >40° N, none of the anthropometric variables consistently exhibited a significant relationship with latitude or mean annual temperature. Significant relationships were returned in five of the analyses in which PI was regressed on mean annual temperature, but not in the other five analyses in which PI was regressed on mean annual temperature. No other significant relationships were identified in the analyses that focused on groups from >40° N. Thus, the supplementary analyses are consistent with the idea that the southern hemisphere subsample did not support Bergmann's rule because the temperature range in the habitable regions of the southern hemisphere is insufficient for thermoregulation-related natural selection to have become the dominant influence on variation in modern human body size.

**Table 5:** Results of regression analyses using stratified 0–40° N subsamples (n = 32). None of the relationships is significant at the Benjamini-Yekutieli adjusted p-value.

		Latitude				Mean annual temperature (°C)			
Subsample		Body mass(kg)	BMI(kg/m^2^)	SA/BM(cm^2^/kg)	PI(kg/m^3^)	Body mass(kg)	BMI(kg/m^2^)	SA/BM(cm^2^/kg)	PI(kg/m^3^)
**1**	**r^2^**	0.007	0.004	0.004	0.002	0.002	0.015	0.012	0.024
	**p-value**	0.658	0.724	0.739	0.825	0.830	0.498	0.548	0.396
**2**	**r^2^**	0.019	0.037	0.038	0.042	0.007	0.002	0.000	0.014
	**p-value**	0.455	0.289	0.288	0.258	0.644	0.832	0.990	0.514
**3**	**rˆ2**	0.035	0.003	0.019	0.002	0.001	0.003	0.006	0.004
	**p-value**	0.307	0.760	0.455	0.804	0.886	0.763	0.665	0.738
**4**	**r^2^**	0.106	0.027	0.067	0.002	0.010	0.006	0.013	0.002
	**p-value**	0.069	0.368	0.151	0.804	0.588	0.684	0.534	0.802
**5**	**r^2^**	0.114	0.048	0.082	0.008	0.019	0.010	0.018	0.003
	**p-value**	0.059	0.230	0.113	0.636	0.453	0.584	0.458	0.772
**6**	**r^2^**	0.023	0.003	0.010	0.000	0.008	0.006	0.010	0.004
	**p-value**	0.409	0.752	0.591	0.987	0.629	0.673	0.588	0.747
**7**	**r^2^**	0.033	0.017	0.022	0.005	0.069	0.071	0.079	0.049
	**p-value**	0.321	0.471	0.415	0.689	0.145	0.139	0.119	0.223
**8**	**r^2^**	0.086	0.001	0.024	0.010	0.020	0.004	0.012	0.000
	**p-value**	0.103	0.839	0.397	0.585	0.442	0.716	0.557	0.925
**9**	**r^2^**	0.023	0.000	0.008	0.012	0.003	0.005	0.008	0.004
	**p-value**	0.413	0.997	0.618	0.557	0.775	0..691	0.637	0.730
**10**	**r^2^**	0.107	0.012	0.048	0.007	0.039	0.048	0.043	0.022
	**p-value**	0.067	0.555	0.229	0.650	0.279	0.231	0.255	0.413

**Table 6:** Results of regression analyses using stratified >40° N subsamples (n = 25). Significant relationships at the Benjamini-Yekutieli adjusted p-value are identified in bold.

		Latitude				Mean annual temperature (°C)			
Subsample		Body mass (kg)	BMI (kg/m^2^)	SA/BM (cm^2^/kg)	PI (kg/m^3^)	Body mass (kg)	BMI (kg/m^2^)	SA/BM (cm^2^/kg)	PI (kg/m^3^)
**1**	**r^2^**	0.055	0.182	0.135	0.199	0.002	0.120	0.057	0.208
	**p-value**	0.257	0.034	0.071	0.025	0.824	0.089	0.252	0.022
**2**	**r^2^**	0.075	0.147	0.153	0.177	0.004	0.063	0.041	0.130
	**p-value**	0.186	0.058	0.053	0.036	0.776	0.226	0.332	0.076
**3**	**r^2^**	0.001	0.103	0.031	0.230	0.001	0.184	0.053	0.388
	**p-value**	0.866	0.119	0.401	0.015	0.914	0.033	0.267	**0.001**
**4**	**r^2^**	0.005	0.079	0.052	0.139	0.019	0.175	0.114	0.285
	**p-value**	0.745	0.174	0.272	0.067	0.511	0.038	0.100	**0.006**
**5**	**r^2^**	0.023	0.214	0.120	0.312	0.010	0.142	0.086	0.218
	**p-value**	0.472	0.020	0.090	**0.004**	0.632	0.063	0.154	0.019
**6**	**r^2^**	0.002	0.075	0.052	0.138	0.005	0.193	0..093	0.369
	**p-value**	0.830	0.185	0.273	0.067	0.743	0.028	0.139	**0.001**
**7**	**r^2^**	0.000	0.075	0.035	0.140	0.009	0.202	0.118	0.321
	**p-value**	0.970	0.186	0.371	0.066	0.647	0.024	0.092	**0.003**
**8**	**r^2^**	0.071	0.128	0.129	0.112	0.012	0.176	0.096	0.186
	**p-value**	0.199	0.079	0.077	0.102	0.597	0.037	0.132	**0.006**
**9**	**r^2^**	0.043	0.080	0.083	0.087	0.011	0.093	0.048	0.163
	**p-value**	0.319	0.171	0.162	0.152	0.621	0.138	0.294	0.046
**10**	**r^2^**	0.065	0.003	0.005	0.087	0.051	0.013	0.001	0.129
	**p-value**	0.220	0.794	0.727	0.152	0.278	0.592	0.871	0.078

It appears, then, that the modification that needs to be made to the hypothesis that modern humans conform to Bergmann's rule concerns latitude and temperature range. Collectively, our analyses suggest that Bergmann's rule holds for modern humans, but only when there are large latitudinal and temperature differences among groups.

What are the minimum latitudinal and temperature ranges over which Bergmann's rule may be observed in modern humans? The analyses discussed in the previous paragraph indicate that the latitudinal range has to be greater than 40° and the temperature range greater than 28°C, but they do not allow us to identify the ranges in question more precisely. With this in mind, we carried out another set of supplementary analyses. In these analyses, we sought significant correlations between the anthropometric variables and the temperature variables at increasingly larger latitudinal scales. We began by extending our randomly-selected, latitudinally-stratified 0–40° N sample to include groups up to 45° N, and then regressed each anthropometric variable on latitude and mean annual temperature again. We continued to increase the latitudinal range of groups at five-degree increments until a significant correlation was observed for each anthropometric variable. The process was repeated nine times to ensure repeatability.

The results of the second set of supplementary analyses are summarized in Table 7. Allowing for the possibility that a couple of the subsamples contain outlier groups, the relationship between body mass and latitude reached significance in the 0–50° N range. The relationship between SA/BM and latitude also reached significance in the 0–50° N range. All the other relationships reached significance in the 0–55° N range. The 0–50° N latitudinal range corresponds to a mean annual temperature range of approximately 30°C while the 0–55° N latitudinal range corresponds to a mean annual temperature range of approximately 32°C. Thus, the second set of supplementary analyses suggest that the minimum latitudinal and temperature ranges over which Bergmann's rule may be observed in modern humans differ depending on the body size variable used but are at least 50° of latitude and 30°C, respectively.

**Table 7:** Results of regression analyses at five-degree latitudinal increments. Each cell shows the number of subsamples (out of ten) in which a significant correlation was observed. MAT  =  mean annual temperature (°C).

	Number of subsamples in which a significant correlation was observed, by latitudinal range					
Analysis	0–45°N	0–50°N	0–55°N	0–60°N	0–65°N	0–70°N
Body mass regressed on latitude	2	9	10	10	10	10
Body mass regressed on MAT	0	7	9	10	10	10
BMI regressed on latitude	0	6	8	9	10	10
BMI regressed on MAT	0	4	8	9	10	10
SA/BM regressed on latitude	0	8	10	10	10	10
SA/BM regressed on MAT	0	5	10	10	10	10
PI regressed on latitude	0	2	2	2	2	8
PI regressed on MAT	0	3	3	3	4	10

This finding has important implications for understanding regional variation in modern human body size. Over the last 60 years, a number of researchers have sought to determine whether regional groups conform to Bergmann's rule. Some of these workers have claimed to find evidence for Bergmann's rule in their samples [20], [23]. Others have failed to find a significant correlation between body size and temperature within regions [17], [19], [22], [33]. Previously it has been assumed that it is the failure to find evidence for the operation of Bergmann's rule that requires explanation, and authors have sought to account for it by appealing to peculiarities of the sample in question. Gilligan and Bulbeck [21], for example, argued that exposure to a non-indigenous diet likely explained their failure to find a significant correlation between body size and latitude among Australia Aborigines. However, the present study suggests a different way of looking at the results of the studies in question. None of the samples that have failed to find evidence for Bergmann's rule encompasses the range of latitudes and/or temperatures that our analyses suggest is necessary to detect the impact of thermoregulation-related natural selection on body size. Thus, the authors' failure to find support for Bergmann's rule is not really surprising. The variation in body size still needs to be explained but the lack of fit with Bergmann's rule does not.

Conversely, the results of the present study cast doubt on Ivanhoe et al. 's [23] and Fukase et al. 's [20] claims to have found evidence that regional body size co-varies with temperature in the manner predicted by Bergmann's rule. Ivanhoe et al. [23] investigated the effects of latitude and nutrition on cranial capacity and a proxy for body size–partial skeletal volume–among indigenous groups along the western coast of North America. They found that both cranial capacity and partial skeletal volume increase with latitude, and concluded from this that Bergmann's rule operated among the groups. Fukase et al. [20] investigated body size variation among Jomon period (ca. 13000–2350 BP) groups in Japan, and concluded that it conformed to Bergmann's rule. Based on our results, unless a region spans more than 50 degrees of latitude or has a range of mean annual temperature in excess of 30°C, it is unlikely that a correlation between body size and latitude/mean annual temperature is reliable evidence for the groups conforming to Bergmann's rule. It is more likely a statistical artifact arising from small sample size or bias in sample selection. Neither the sample used by Ivanhoe et al. [23] nor the one employed by Fukase et al. [20] meets the two criteria set out above. The groups included in Ivanhoe et al. 's [23] sample are distributed across approximately 34 degrees of latitude. The groups in Fukase et al. 's [20] sample are even more narrowly distributed. They span no more than 15 degrees of latitude. Thus, our study suggests that Ivanhoe et al. 's [23] and Fukase et al. 's [20] claims should be treated with skepticism.

Three possibilities for further research suggest themselves. The first concerns female body size variation. To reiterate, in the study reported here we focused on male body size in order to control for the potentially confounding effects of sexual dimorphism. There is reason to think that our findings hold for females as well as males. Perhaps most significantly, in previous global-scale tests of the hypothesis that modern humans conform to Bergmann's rule female samples have yielded similar results to male samples [8], [9], [12]. However, it would be useful to repeat the analyses reported here with female data to ensure that the minimum ranges of latitude and temperature over which Bergmann's rule may be observed are the same in females and males. A second possibility for further research concerns temperature. As we noted earlier, most authors have used latitude and/or mean annual temperature when investigating Bergmann's rule in humans [12], [17]. However, it has been suggested that temperature extremes may be the target of thermoregulation-related natural selection [34]. Given the results of the present study, this hypothesis seems worth revisiting. If it is correct, regressing modern human body size on minimum and maximum annual temperatures should yield higher effect sizes than regressing it on latitude and mean annual temperature. Lastly, as we explained earlier, there is no consensus about which anthropometric variable should be used when investigating Bergmann's rule in modern humans. In our study we dealt with this uncertainty by using four different variables to capture body size. We had expected to find some of the variables to be consistently more strongly influenced by temperature than others, but our results were otherwise: the anthropometric variable that had the strongest association with the temperature variables differed by analysis (Tables 1–6). This suggests that the manner in which thermoregulation-related natural selection can be expected to impact different body size variables requires further work.

## Conclusions

In this paper we have reported the results of a study in which we revisited a long-standing anthropological “fact”–namely that modern human body size increases as temperature decreases and therefore conforms to Bergmann's rule. We did so because the main studies that have reported that modern humans conform to Bergmann's rule have employed samples that contain a disproportionately large number of warm-climate and northern hemisphere groups. In our study, we used latitudinally-stratified and hemisphere-specific samples to re-assess the relationship between modern human body size and temperature. We found that when groups from north and south of the equator were analyzed together, Bergmann's rule was supported. However, when groups were analysed by hemisphere, Bergmann's rule was only supported in the northern hemisphere. In the course of exploring these results further, we found that the difference between our northern and southern hemisphere subsamples is due to the limited latitudinal and temperature range in the latter subsample. Thus, our study suggests that modern humans do conform to Bergmann's rule but only when there are major differences in latitude and temperature among groups. Specifically, groups must span more than 50 degrees of latitude and/or more than 30°C for it to hold. This finding has important implications for work on regional variation in body size and how it relates to Bergmann's rule. Perhaps most notably it suggests that recent claims to have found evidence for the operation of Bergmann's rule in regional samples of modern humans should be viewed with scepticism [20], [23].

## Supporting Information

Table S1 **Groups in sample.**
(DOCX)

## References

[1] Bergmann C (1847) Ueber die verhältnisse der wärmeökonomie der thiere zuihrer Grösse. Göttinger Studien 3: 595–708.

[2] Gladwin T (1949) Climate and anthropology. Am Anthropol 49: 601–611. 10.1525/aa.1947.49.4.02a00070 20268090

[3] Schreider E (1950) Geographical distribution of the body-weight/body-surface ratio. Nature 165: 286. 10.1038/165286b0 15410342

[4] Schreider E (1951) Anatomical factors of body-heat regulation. Nature 167: 823–824. 14833431 10.1038/167823a0

[5] Newman MT (1953) The application of ecological rules to the racial anthropology of the Aboriginal New World. Am Anthropol 55: 311–327.

[6] Hanna JM, Little MA, Austin DA (1989) Climate and Physiology. In Little MA, Haas JE, editors. Human Population Biology: a transdisciplinary science. Oxford: Oxford University Press. 132–151.

[7] Ruff CB (1994) Morphological adaptation to climate in modern and fossil hominids. Am J Phys Anthropol 37: 65–107.

[8] Wells JCK (2012) Ecogeographical associations between climate and human body composition: analyses based on anthropometry and skinfolds. Am J Phys Anthropol 47: 169–186. 10.1002/ajpa.21591 22212891

[9] Roberts DF (1978) Climate and Human Variability 2^nd^ Ed. Menlo Park: Cummings Publishing Co. Inc. 123 p.

[10] Walter H (1976) Körperbauform und klima: kritische Überlegungen zur Übertragbarkeit der Bergmannschen regel auf den menschen. Z Morphol Anthropol 67: 241–263. 1007391

[11] Crognier E (1981) Climate and anthropometric variations in Europe and the Mediterranean area. Ann Hum Biol 8: 99–107. 7247349 10.1080/03014468100004841

[12] Katzmarzyk PT, Leonard WR (1998) Climatic influences on human body size and proportions: Ecological adaptations and secular trends. Am J Phys Anthropol 106: 483–503. 9712477 10.1002/(SICI)1096-8644(199808)106:4<483::AID-AJPA4>3.0.CO;2-K

[13] Stinson S, Bogin B, Huss-Ashmore R, O'Rourke D (2000) Human Biology: An Evolutionary and Biocultural Perspective. New York: Wiley-Liss. 639 p.

[14] Lewin R, Foley RA (2004) Principles of Human Evolution 2^nd^ Ed. Malden: Wiley-Blackwell. 576 p.

[15] Jurmain R, Kilgore L, Trevathan W (2012) Essentials of Physical Anthropology (8^th^ Edition).Belmont: Wadsworth. 448 p.

[16] Stanford C, Allen JS, Anton SC (2012) Exploring Biological Anthropology: The Essentials 3^rd^ Ed. Upper Saddle River: Prentice Hall. 480 p.

[17] Roberts DF (1953) Body weight, race and climate. Am J Phys Anthropol 11: 533–558. 13124471 10.1002/ajpa.1330110404

[18] Beall CM, Goldstein MC (1992) High prevalence of excess fat and central fat patterning among Mongolian pastoral nomads. Am J Phys Anthropol 4: 747–756. 10.1002/ajhb.1310040606 28524633

[19] Hiernaux J, Froment A (1976) Correlations between anthropo-biological and climatic variables in sub-Saharan Africa: Revised estimates. Hum Biol 48: 757–767. 1017818

[20] Fukase H, Wakebe T, Tsurumoto T, Saiki K, Fujita M, et al (2012) Geographic Variation in Body Form of Prehistoric Jomon Males in the Japanese Archipelago: Its Ecogeographic Implications. Am J Phys Anthropol 149: 125–135. 22791466 10.1002/ajpa.22112

[21] Gilligan I, Bulbeck D (2007) Environment and morphology in Australian Aborigines: a reanalysis of the Birdsell Database. Am J Phys Anthropol 134: 75–91. 17568440 10.1002/ajpa.20640

[22] Stinson S (1990) Variation in body size and shape among South American Indians. Am J Hum Biol 2: 37–51. 28520262 10.1002/ajhb.1310020105

[23] Ivanhoe F, Chu PW, Bennyhoff JA (1998) Archaeological Amerindian and Eskimo cranioskeletal size variation along coastal Western North America: relation to climate, the reconstructed diet high in marine animal foods, and demographic stress. Int J Osteoarchaeol 8: 135–179.

[24] Eveleth PB, Tanner JM (1976) Worldwide Variations in Human Growth. Cambridge: Cambridge University Press. 397 p.

[25] Eveleth PB, Tanner JM (1990) Worldwide Variations in Human Growth 2^nd^ Ed. Cambridge: Cambridge University Press. 497 p.

[26] Dubois D, Dubois EF (1916) A formula to estimate the approximate surface area if height and weight be known. Arch Intern Med 17: 863–71.

[27] Cross AG, Collard M (2011) Estimating surface area in fossil hominins. PLoS ONE 6: e16107 doi:10.1371/journal.pone.0016107 21249191 10.1371/journal.pone.0016107 PMC3020943

[28] New M, Hulme M, Jones P (1999) Representing twentieth-century space-time climate variability. Part I: Development of a 1961–90 mean monthly terrestrial climatology. J Clim 12: 829–856.

[29] Quinlan RJ (2007) Human parental effort and environmental risk. Proc Biol Sci 274: 121–125. 17134996 10.1098/rspb.2006.3690 PMC1679876

[30] Motulsky H, Christopoulous A (2003) Fitting models to biological data using linear and nonlinear regression. A practical guide to curve fitting. San Diego: GraphPad Software Inc. 351 p.

[31] Benjamini Y, Yekutieli D (2001) The control of false discovery rate under dependency. Ann Stat 29: 1165–1188.

[32] Narum SR (2006) Beyond Bonferroni: Less conservative analyses for conservation genetics. Conserv Genet 7: 783–787.

[33] Temple DH, Matsumura H (2011) Do body proportions among Jomon foragers from Hokkaido conform to ecogeographic expectations? Evolutionary implications of body size and shape among northerly hunter-gatherers. Int J Osteoarchaeol 21: 268–282.

[34] Newman RW, Munro EH (1955) The relation of climate and body size in U.S. males. Am J Phys Anthropol 13: 1–17. 14361654 10.1002/ajpa.1330130102

