# Supplementary material for: A Reassessment of Bergmann's Rule in Modern Humans
Source: PLoS One. 2013 Aug 28;8(8):e72269. doi: 10.1371/journal.pone.0072269 (PMC3756069; doi:10.1371/journal.pone.0072269)
Supplement: Table S1 — Groups in sample. (DOCX) [file pone.0072269.s001.docx]

**Supporting information for Foster and Collard’s “A reassessment**

**of Bergmann’s rule in modern humans”**

**Table S1. Groups in sample. Lat = latitude. Long = longitude. A few populations have more than one entry. This is because an author listed more than one body mass value for a population without explanation, or because different authors provided different body masses for a population. In these cases, we assumed that the different body mass values pertain to different groups of the same population. We elected not to choose between the body mass values and to simply avoid the duplicates in the creation of the stratified subsamples.**

| **Group** | **Source** | **Lat** | **Long** | **Notes** |
| --- | --- | --- | --- | --- |
| !Kung | Winkler and Kirchengast (1994) | -20.30 | 18.00 |  |
| Ache | Walker and Hill (2003) | -24.16 | 305.51 |  |
| Achuar | Patton (1996) | -1.87 | 283.22 |  |
| Ainu | Picon-Reategui et al. (1979) | 43.77 | 142.37 |  |
| Aita | Eveleth and Tanner (1990) | -8.87 | 142.90 |  |
| Akka | Roberts (1953) | 2.39 | 18.02 |  |
| Andamanese 1 | Man (1882) | 12.50 | 92.75 |  |
| Andamanese 2 | Portman (1896) | 12.50 | 92.75 |  |
| Annamites | Roux (1905) | 22.00 | 105.00 |  |
| Apache | Eveleth and Tanner (1976) | 33.92 | 249.83 |  |
| Arequipa | Hurtado (1928) | -16.40 | 288.46 |  |
| Assiniboine | Eveleth and Tanner (1976) | 48.48 | 251.23 |  |
| Auyana | Littlewood (1972) | -6.57 | 145.72 |  |
| Awa | Littlewood (1972) | -6.60 | 145.73 |  |
| Aymara | Beall et al. (1997) | -16.50 | 291.85 |  |
| Badjoue | Roberts (1953) | 3.17 | 13.80 |  |
| Baegu | Eveleth and Tanner (1990) | -9.00 | 161.00 |  |
| Bahutu 1 | Roberts (1953) | -2.07 | 29.76 |  |
| Bahutu 2 | Roberts (1953) | -2.07 | 29.76 |  |
| Bakiga | Roberts (1953) | 0.92 | 31.17 |  |
| Banjarese | Roberts (1953) | -3.33 | 114.59 |  |
| Bantu | Wyndham et al. (1964a) | -30.00 | 22.00 |  |
| Batutsi 1 | Roberts (1953) | -1.59 | 29.91 |  |
| Batutsi 2 | Roberts (1953) | -1.59 | 29.91 |  |
| Baya | Roberts (1953) | 4.37 | 18.58 |  |
| Beaver, Cree, and Slave | Lee and Birkbeck (1977) | 56.25 | 120.85 |  |
| Bedik | Gomila (1971) | 13.00 | 347.75 |  |
| Belgian students and workers | Eveleth and Tanner (1976) | 50.85 | 4.35 |  |
| Bella | Froment and Hiernaux (1984) | 14.68 | 359.54 |  |
| Bengal Indians | Roberts (1953) | 23.71 | 90.41 |  |
| Bira of rain forest | Sporcq (1975) | 0.42 | 28.63 |  |
| Bira of savannah | Sporcq (1975) | 1.63 | 30.08 |  |
| Blackfeet | Eveleth and Tanner (1976) | 48.56 | 246.99 |  |
| Bombay Indians 1 | Roberts (1953) | 19.02 | 72.86 |  |
| Bombay Indians 2 | Roberts (1953) | 19.02 | 72.86 |  |
| Brahmin Indians | Roberts (1953) | 19.02 | 72.86 |  |
| Bulang | Chen (1992) | 25.00 | 102.00 |  |
| Bulgarians | Eveleth and Tanner (1976) | 42.70 | 23.33 |  |
| Bulgars | Roberts (1953) | 42.70 | 23.33 |  |
| Buryats | Kozlov (2007) | 51.83 | 107.60 |  |
| Bwaba | Froment and Hiernaux (1984) | 11.80 | 356.72 |  |
| Caingang | Eveleth and Tanner (1976) | -27.50 | 307.50 |  |
| Car Nicobarese | Ganguly (1976) | 9.17 | 92.78 |  |
| Cashinahua | Eveleth and Tanner (1976) | -10.00 | 289.00 |  |
| Central Australian Aborigines 1 | Abbie (1975) | -22.74 | 131.86 |  |
| Central Australian Aborigines 2 | Roberts (1953) | -22.74 | 131.86 |  |
| Central Australian Aborigines 3 | Roberts (1953) | -22.74 | 131.86 |  |
| Central Chinese 1 | Roberts (1953) | 30.66 | 104.06 |  |
| Central Chinese 2 | Roberts (1953) | 30.66 | 104.06 |  |
| Chaamba | Coblentz (1968) | 34.57 | 3.78 |  |
| Chachi | Stinson (1989) | 0.97 | 280.96 |  |
| Chaco | Eveleth and Tanner (1976) | -26.59 | 299.05 |  |
| Chamula Indians | Leche (1936) | 16.78 | 267.32 |  |
| Choctaw 1 | Collins (1928) | 35.47 | 262.48 |  |
| Choctaw 2 | Roberts (1953) | 35.47 | 262.48 |  |
| Chol | Gould (1946) | 17.30 | 267.68 |  |
| Chowrite | Ganguly (1976) | 8.45 | 93.03 |  |
| Chung Chong | Roberts (1953) | 36.35 | 127.38 | Also referred to as the Chuncheong in the source(s) consulted. |
| Cornish | Roberts (1953) | 50.26 | 354.95 |  |
| Cypriotes | Angel (1972) | 34.67 | 32.90 |  |
| Czech | Prokopec (1977) | 50.08 | 14.47 |  |
| Danes | Holloway (1980) | 55.68 | 12.57 |  |
| Dogon | Froment and Hiernaux (1984) | 15.28 | 358.30 |  |
| Donse Mossi | Froment and Hiernaux (1984) | 11.43 | 357.83 |  |
| Durban Zulus | Slome et al. (1960) | -29.90 | 31.00 |  |
| Dutch | Eveleth and Tanner (1976) | 52.37 | 4.89 |  |
| Edinburgh Scottish | Roberts (1953) | 55.95 | -3.19 |  |
| English | Roberts (1953) | 52.48 | 358.11 |  |
| Evenks | Leonard, et al. (1994) | 62.00 | 94.68 |  |
| Filipino | Roberts (1953) | 14.58 | 120.97 |  |
| Finns | Ruff et al. (2005) | 65.56 | 28.25 |  |
| Fort Severn Cree | Hurlich and Steegmann (1979) | 56.01 | -87.59 |  |
| Fukus Japanese | Shapiro (1939) | 37.50 | 140.25 |  |
| Fulero | Hiernaux (1956) | -3.41 | 29.15 |  |
| Gadsup | Littlewood (1972) | -6.35 | 145.93 |  |
| Gagou | Roberts (1953) | 6.15 | -5.95 | Also referred to as the Gagu in the source(s) consulted. |
| Glasgow Scottish | Roberts (1953) | 55.87 | 355.74 |  |
| Goranes | Coblentz (1968) | 17.25 | 22.00 | Also referred to as the Bideyat in the source(s) consulted. |
| Greeks | Roberts (1953) | 37.98 | 23.73 |  |
| Greenland Eskimos | Bjerregaard et al. (2003) | 67.93 | -51.42 |  |
| Gurmanche | Froment and Hiernaux (1984) | 12.80 | 0.70 |  |
| Gyeli | Froment (1989) | 2.27 | 9.86 |  |
| Hadza | Hiernaux and Boedhi Hartono (1980) | -4.00 | 35.08 |  |
| Hama | Roberts (1953) | 61.00 | 24.47 | Also referred to as the Hame in the source(s) consulted. |
| Hani | Chen (1992) | 25.00 | 102.00 |  |
| Havu | Hiernaux (1956) | -2.10 | 28.92 |  |
| Hawaiians | Roberts (1953) | 21.31 | -157.86 |  |
| Hiroshima Japanese | Shapiro (1939) | 34.40 | 132.45 |  |
| Hoklo | Roberts (1953) | 25.09 | 121.56 |  |
| Hong Kong Chinese 1 | Roberts (1953) | 22.40 | 114.11 |  |
| Hong Kong Chinese 2 | Roberts (1953) | 22.40 | 114.11 |  |
| Hopi | Gabel (1949) | 35.88 | 249.36 |  |
| Humu | Hiernaux (1956) | 0.38 | 29.90 |  |
| Hunde | Hiernaux (1956) | -1.29 | 29.13 |  |
| Hungarians | Eveleth and Tanner (1976) | 47.50 | 19.04 |  |
| Hutu de l'Urundi | Hiernaux (1956) | -3.38 | 29.36 |  |
| Hutu du Ruanda | Hiernaux (1956) | -2.07 | 29.76 |  |
| Hwanhai | Roberts (1953) | 38.04 | 125.71 | Also referred to as the Hwanghae in the source(s) consulted. |
| Hyderabad Indians | Roberts (1953) | 17.39 | 78.49 |  |
| Icelandic | Roberts (1953) | 64.13 | 24.93 |  |
| Igloolik Inuits | de Pena (1971) | 69.40 | -81.80 |  |
| Iroquois | Gould (1869) | 43.17 | 80.25 |  |
| Ituri Pygmies 1 | Cavalli-Sforza (1986) | 1.85 | 29.96 | Also referred to as the Efe Pygmies in the source(s) consulted. |
| Ituri Pygmies 2 | Roberts (1953) | 1.85 | 29.96 | Also referred to as the Efe Pygmies in the source(s) consulted. |
| Japanese | Roberts (1953) | 35.69 | 139.69 |  |
| Javanese | Hastuti (2009) | -7.78 | 110.37 |  |
| Javanese 1 | Roberts (1953) | -6.21 | 106.85 |  |
| Javanese 2 | Roberts (1953) | -6.21 | 106.85 |  |
| Jinuo | Chen (1992) | 25.00 | 102.00 |  |
| Kainuu | Roberts (1953) | 64.23 | 27.73 |  |
| Karjalans | Roberts (1953) | 63.75 | 33.98 | Also referred to as the Karelians in the source(s) consulted. |
| Karkar Islanders | Harvey (1974) | -4.55 | 146.01 |  |
| Kavango | Kirchengast and Winkler (1995) | -17.93 | 19.77 |  |
| Kazaks | Roberts (1953) | 51.18 | 71.45 |  |
| Khalkha Mongols | Eveleth and Tanner (1976) | 47.92 | 106.91 |  |
| Kikuyu 1 | Roberts (1953) | 1.27 | 36.80 |  |
| Kikuyu 2 | Roberts (1953) | 1.27 | 36.80 |  |
| Kirdi 1 | Roberts (1953) | 10.47 | 13.60 |  |
| Kirdi 2 | Roberts (1953) | 10.47 | 13.60 |  |
| Kirghiz | Roberts (1953) | 42.87 | 74.61 | Also referred to as the Kyrgyz in the source(s) consulted. |
| Kokologo Mossi | Froment and Hiernaux (1984) | 12.19 | 358.11 |  |
| Koma | Froment (1989) | 9.00 | 12.87 |  |
| Komi | Kozlov (2007) | 63.15 | 55.83 |  |
| Komi-Permyaks | Kozlov (2007) | 58.00 | 56.23 |  |
| Korean | Roberts (1953) | 37.57 | 126.98 |  |
| Kota | Kumar (2000) | 11.42 | 76.50 |  |
| Kuben-Kran-Kegn | da Rocha and Salzano (1972) | -8.17 | 307.87 |  |
| Kurumba | Kumar (2000) | 11.42 | 76.50 |  |
| Kwaio | Eveleth and Tanner (1990) | -9.00 | 161.00 |  |
| Kyong Kwi | Roberts (1953) | 37.26 | 127.03 | Also referred to as the Kyungki or the Gyeonggi in the source(s) consulted. |
| Kyushu Japanese | Shapiro (1939) | 33.58 | 130.40 | Also referred to as the Fukuoka Japanese in the source(s) consulted. |
| Lahaul Kanets | Singh et al. (2008) | 32.58 | 77.00 |  |
| Lake Eyre Basin | Fenner (1936) | -28.00 | 137.50 |  |
| Lapps | Auger et al. (1980) | 68.90 | 27.03 |  |
| Lau | Eveleth and Tanner (1990) | -9.00 | 161.00 |  |
| Lauans | Eveleth and Tanner (1976) | -17.28 | 181.02 |  |
| Leipzig students | Eveleth and Tanner (1976) | 51.34 | 12.37 |  |
| Londoners 1 | Roberts (1953) | 51.51 | 0.13 |  |
| Londoners 2 | Roberts (1953) | 51.51 | 0.13 |  |
| Luba | Hiernaux (1972) | -11.68 | 27.45 |  |
| Lufa | Harvey (1974) | -6.35 | 145.34 |  |
| Madras Indians | Roberts (1953) | 13.06 | 80.25 |  |
| Mahratta Indians | Roberts (1953) | 19.02 | 72.86 | Also referred to as Marathi Indians in the source(s) consulted. |
| Maku | Milton (1983) | 0.02 | 291.37 |  |
| Mal Paharia | Roberts (1953) | 22.78 | 86.21 |  |
| Malavar | Fawcett (1903) | 8.73 | 77.70 |  |
| Malays | Roberts (1953) | 3.14 | 101.69 |  |
| Malebe | Froment and Hiernaux (1984) | 14.68 | 359.54 |  |
| Mam | Diaz et al. (1991) | 15.32 | 268.53 |  |
| Mande | Roberts (1953) | 12.65 | -8.00 |  |
| Mansi | Kozlov (2007) | 61.03 | 69.09 |  |
| Maori | Roberts (1953) | -36.85 | 174.77 |  |
| Mapuche | Pi-Suner (1933) | -38.78 | 286.60 |  |
| Marsa Matrouh | Ćwirko-Godycki (1961) | 31.35 | 27.23 |  |
| Masai | Roberts (1953) | -3.00 | 37.50 |  |
| Massawa | Roberts (1953) | -4.35 | 152.04 |  |
| Maxqong Bushmen | Wyndham et al. (1964b) | -23.25 | 22.25 |  |
| Maya | Steggerda (1932) | 19.30 | 271.00 |  |
| Maya 1 | Roberts (1953) | 19.30 | 271.00 |  |
| Maya 2 | Roberts (1953) | 19.30 | 271.00 |  |
| Maya 3 | Roberts (1953) | 19.30 | 271.00 |  |
| Maya 4 | Roberts (1953) | 19.30 | 271.00 |  |
| Mbuba | Hiernaux (1956) | -4.71 | 15.09 |  |
| Mbya-Guarani | Oyenhart, et al. (2003) | -27.10 | 305.10 |  |
| Mekhadma | Coblentz (1968) | 34.65 | 5.48 |  |
| Mekranoti | da Rocha and Salzano (1972) | -8.67 | 306.00 |  |
| Melanesians | Eveleth and Tanner (1990) | -18.17 | 178.45 |  |
| Melville and Bathurst Islands Aborigines | Howells (1937) | -11.64 | 130.69 |  |
| Mentawei | Roberts (1953) | -2.70 | 100.13 | Also referred to as the Mentawai in the source(s) consulted. |
| Miao | Roberts (1953) | 28.23 | 112.94 |  |
| Miskito | de Stefano and Jenkins (1972) | 13.88 | 275.42 |  |
| Murrayians | Birdsell (1967) | -35.12 | 139.27 |  |
| Mvae | Froment (1989) | 2.27 | 9.86 |  |
| Naga | Roberts (1953) | 25.91 | 93.73 |  |
| Nagovisi | Eveleth and Tanner (1990) | -6.00 | 155.00 |  |
| Nasioi | Eveleth and Tanner (1990) | -6.00 | 155.00 |  |
| Navaho 1 | Roberts (1953) | 36.10 | 249.78 |  |
| Navaho 2 | Sandstead et al. (1956) | 36.10 | 249.78 |  |
| New Ireland | Schlaginhaufen (1964) | -3.33 | 152.00 |  |
| Nootka | Birkbeck et al. (1971) | 49.28 | 126.07 |  |
| North Australian Aborigines | Macho and Freedman (1987) | -13.32 | 133.58 |  |
| North Chinese 1 | Roberts (1953) | 40.84 | 111.75 |  |
| North Chinese 2 | Roberts (1953) | 40.84 | 111.75 |  |
| North Tuchone | Lee and Birkbeck (1977) | 61.98 | 132.45 |  |
| Nyanga | Hiernaux (1956) | -1.42 | 28.06 |  |
| Nyuturu | Roberts (1953) | -4.82 | 34.74 |  |
| Ojibwa | Katzmarzyk and Malina (1999) | 47.00 | 280.00 |  |
| Ontenu | Littlewood (1972) | -6.35 | 145.83 |  |
| Ontong Java | Eveleth and Tanner (1990) | -5.33 | 159.50 |  |
| Orochee | Roberts (1953) | 48.48 | 135.09 | Also referred to as the Oroch or Orochi in the source(s) consulted. |
| Ostiaks | Kozlov (2007) | 61.03 | 69.09 | Also referred to as the Khanty in the source(s) consulted. |
| Pahira | Basu (1969) | 22.88 | 86.00 |  |
| Parisians | Roberts (1953) | 48.86 | 2.35 |  |
| Pekin Chinese 1 | Roberts (1953) | 39.90 | 116.41 | Also referred to as the Peking Chinese in the source(s) consulted. |
| Pekin Chinese 2 | Roberts (1953) | 39.90 | 116.41 | Also referred to as the Peking Chinese in the source(s) consulted. |
| Pjonjan | Roberts (1953) | 39.03 | 125.75 | Also referred to as the Pyongyang in the source(s) consulted. |
| Pohjanmaa | Roberts (1953) | 63.17 | 22.00 |  |
| Polish | Eveleth and Tanner (1976) | 50.06 | 19.94 |  |
| Quechua | Eveleth and Tanner (1976) | -14.48 | 289.36 |  |
| Rama | de Stefano and Jenkins (1972) | 12.15 | 275.78 |  |
| Rega | Hiernaux (1956) | -6.87 | 27.57 |  |
| Reguibat | Coblentz (1968) | 26.70 | 354.00 |  |
| Rimaibe | Froment and Hiernaux (1984) | 14.68 | 359.54 |  |
| Romanian | Eveleth and Tanner (1976) | 44.43 | 26.10 |  |
| Rural Venda | Eveleth and Tanner (1976) | -22.95 | 30.48 |  |
| Salloum | Ćwirko-Godycki (1961) | 25.15 | 31.57 |  |
| Samoyedes | Kozlov (2007) | 66.55 | 66.60 | Also referred to as the Nenets in the source(s) consulted. |
| Sandawe | Roberts (1953) | -4.90 | 35.78 |  |
| Santa Lucia | Hurtado (1928) | -15.69 | 289.39 |  |
| Santal | Roberts (1953) | 24.26 | 87.25 | Also referred to as the Santhal in the source(s) consulted. |
| Sara | Crognier (1972) | 10.50 | 17.90 |  |
| Sauria Paharia | Roberts (1953) | 24.26 | 87.25 |  |
| Savonians | Roberts (1953) | 62.89 | 27.68 |  |
| Seminole | Eveleth and Tanner (1976) | 26.74 | 279.38 |  |
| Senoi 1 | Roberts (1953) | 3.14 | 101.69 |  |
| Senoi 2 | Roberts (1953) | 3.14 | 101.69 |  |
| Sherpas | Sloan and Masali (1978) | 27.52 | 86.60 |  |
| Shi | Hiernaux (1956) | -2.51 | 28.86 |  |
| Shipibo | Hanna and Baker (1974) | -7.90 | 285.25 |  |
| Shu | Hiernaux (1956) | 0.20 | 29.28 |  |
| Sicilians | Roberts (1953) | 38.12 | 13.37 |  |
| Sidi Barrany | Ćwirko-Godycki (1961) | 31.60 | 25.92 |  |
| Siwah | Ćwirko-Godycki (1961) | 29.17 | 25.67 |  |
| Slave | Lee and Birkbeck (1977) | 60.05 | 128.91 |  |
| South Chinese 1 | Roberts (1953) | 22.82 | 108.37 |  |
| South Chinese 2 | Roberts (1953) | 22.82 | 108.37 |  |
| Southeast English | Roberts (1953) | 51.51 | 0.13 |  |
| Southern Nicobarese | Ganguly (1976) | 7.40 | 93.67 |  |
| St. Lawerence Island Inuit | Harper et al. (1984) | 63.42 | 170.40 |  |
| Subtiava | de Stefano and Jenkins (1972) | 12.44 | 273.12 |  |
| Sudanese 1 | Roberts (1953) | -6.21 | 106.85 | These are Sudanese people living in Indonesia. |
| Sudanese 2 | Roberts (1953) | -6.21 | 106.85 | These are Sudanese people living in Indonesia. |
| Sumo | de Stefano and Jenkins (1972) | 13.00 | 276.25 |  |
| Swaga | Hiernaux (1956) | -6.50 | 24.76 |  |
| Szechwan Chinese | Roberts (1953) | 30.66 | 104.06 |  |
| Tairora | Littlewood (1972) | -6.35 | 145.83 |  |
| Tembo | Hiernaux (1956) | -2.10 | 28.92 |  |
| Terressan | Ganguly (1976) | 8.25 | 93.22 |  |
| Tibesti Toubous | Coblentz (1968) | 21.50 | 17.50 |  |
| Tibetans | Beall et al. (1997) | 29.65 | 91.14 |  |
| Toda | Gates (1961) | 11.42 | 76.50 |  |
| Trio and Wajana | Glanville and Geerdink (1972) | 3.63 | 304.33 |  |
| Tsilhqot'in | Birkbeck et al. (1971) | 52.47 | 125.32 |  |
| Tukanoan | Dufour (1981) | 1.00 | 290.40 |  |
| Tusti de l'Urundi | Hiernaux (1956) | -3.38 | 29.36 |  |
| Tutsi du Ruanda-Urundi | Hiernaux (1956) | -1.59 | 29.91 |  |
| Twa Pygmies | Roberts (1953) | -1.91 | 29.56 | Also referred to as the Batwa Pygmies in the source(s) consulted. |
| Txukahamae | da Rocha and Salzano (1972) | -10.33 | 306.92 |  |
| Ulawa | Eveleth and Tanner (1990) | -9.77 | 161.95 |  |
| Urban Venda | Eveleth and Tanner (1976) | -22.95 | 30.48 |  |
| Uusimaa | Roberts (1953) | 60.17 | 24.94 |  |
| Varsais | Roberts (1953) | 60.45 | 22.27 |  |
| Victoria River | Howells (1937) | -15.20 | 129.72 |  |
| Wainwright Inuits | Eveleth and Tanner (1976) | 70.64 | 200.17 |  |
| Weagamow Ojibwa | Hurlich and Steegmann (1979) | 52.89 | 268.63 |  |
| Xavante | Neel et al. (1964) | -13.50 | 308.58 |  |
| Yalata | Macho and Freedman (1987) | -31.38 | 131.62 |  |
| Yamaguchi Japanese | Shapiro (1939) | 35.22 | 134.80 |  |
| Yambasa | Roberts (1953) | 4.52 | 11.25 |  |
| Yaqui | Roberts (1953) | 29.17 | 249.05 |  |
| Yassa | Froment (1989) | 2.27 | 9.86 |  |
| Yugosalvian Gypsies | Eveleth and Tanner (1976) | 46.75 | 16.25 |  |
| Zuni | Gabel (1949) | 35.07 | 251.15 |  |

**Literature cited in Table S1**

Abbie AA (1975) Metric characteristics of adult Aborigines. Stud Phys Anth 2: 76-103.

Angel JL (1972) Ecology and population in the eastern Mediterranean. World Archaeol 4: 88-105.

Auger F, Jamison P, Balslev-Jorgensen J, Lewin T, de Pena J, Skrobak-Kaczynski J (1980) Anthropometry of circumpolar populations. In: Milan FA, editor. Biology of Circumpolar Populations. Cambridge: Cambridge University Press. 213–255.

Basu A (1969) The Pahira: a population genetical study. Am J Phys Anthropol 31: 399-416.

Beall CM, Strohl KP, Blangero J, Williams-Blangero S, Almasy LA, Decker MJ, Worthman CM, Goldstein MC, Vargas E, Villena M, Soria R, Alarcon AM, Gonzales C (1997) Ventilation and hypoxic ventilatory response of Tibetan and Aymara high altitude natives. Am J Phys Anthropol 104: 427-447.

Birdsell, JB (1967) Preliminary data on the trihybrid origin of the Australian Aborigines. Archaeol Phys Anthropol Oceania 2: l00-155.

Birkbeck JA, Lee M, Myers GS, Alfred BM (1971) Nutritional status of British Columbia Indians II: anthropometric measurements, physical and dental examinations at Ahousat and Anaham. C J Public Health 62: 403–414.

Bjerregaard P, Curtis T, Borch-Johnsen K, Mulvad G, Becker U, Andersen S, Backer V (2003) Inuit health in Greenland: a population survey of life style and disease in Greenland and among Inuit living in Denmark. Int J Circumpol Heal (Suppl. 1): 3-79.

Cavalli-Sforza LL (1986) Anthropometric data. In: Cavali-Sforza LL, editor. African Pygmies. New York: Academic Press. 81–93.

Chen G, Lin J (1992) Ren lei xue yu ying yong / Zhongguo ren lei xue xue hui bian. Shanghai: Xue lin chu ban she. 137 p.

Clegg EG (1989) The growth of Melanesian and Indian children in Fiji. Ann Hum Biol 16: 507–528.

Coblentz A (1968) Les liaisons des caractères métriques de la main**.** B Mem Soc Anthro Par 3: 331-345.

Collins HB Jr (1928) Additional anthropometric observations on the Choctaw. Am J Phys Anthropol 11: 353-355.

Crognier É (1972). Adaptation morphologique d'une population africaine au biotope tropical, les Sara du Tchad (Doctoral dissertation). Paris: Universite de Paris.

Ćwirko-Godycki M (1961) Publications of the joint Arabic-Polish anthropological expedition 1958/1959: Part I, anthropological measurements of population in the United Arab Republic (Egypt-West Desert). Warsaw: Poznam. 575 p.

da Rocha FJ, Salzano FM (1972) Anthropometric studies in Brazilian Cayapo Indians. Am J Phys Anthropol 36: 95-101.

de Pena J (1972) Growth and development. In: Hughes DR, editor. IBP Annual Report No. 4, Human Adaptability Project, University of Toronto Anthropological Series 11: 47-69.

de Stefano GF, Jenkins JM (1972) Richerche de anthropologia biologica su populazioni nicaraguensi. Rev Anthropol 58*:*289–302.

Diaz ET, González-Cossió T, Rivera J, Immink MDC, Mendoza RD, Flores CR (1991) Body composition estimates using different measurement techniques in a sample of highland subsistence farmers in Guatemala. Am J Hum Biol 3: 525-530.

Dufour DL (1981) Household variation in a population of tropical forest horticulturalists (Doctoral dissertation). Binghamptom: State University of New York at Binghamton.

Eveleth PB, Tanner JM (1976) Worldwide Variations in Human Growth. Cambridge: Cambridge University Press. 397 p.

Eveleth PB, Tanner JM (1990) Worldwide Variations in Human Growth, 2nd Ed. Cambridge: Cambridge University Press. 497 p.

Fawcett F (1903) The Kondayamkottai Maravars, a Dravidian tribe of Tinnevelly, Southern India. J R Anthropol Inst G 33: 57-65.

Fenner FJ (1936) Adelaide University field anthropology, Central Australia: no. 13 – Anthropomorphic observations on South Australian Aborigines of the Diamantina and Cooper Creek regions. T Roy Soc South Aust 60: 46-54.

Friedlaender JS, Rhoads JG (1987) Longitudinal anthropometric changes in adults and adolescents. In: Friedlaender JS, editor. The Solomon Islands Project: A Long Term Study of Health, Human Biology and Culture Change. Oxford: Clarendon Press. 283–306.

Frisancho AR, Baker PT (1970) Altitude and growth: A study of the patterns of physical growth of a high altitude Peruvian Quechua population. Am J Phys Anthropol 32: 279-292.

Froment AJ (1989) Body morphology and the savanna – forest transition: a West African example. Int J Anthropol 4: 61-74.

Froment AJ, Hiernaux J (1984) Climate-associated anthropometric variation between populations of the Niger bend. Ann Hum Biol 11: 189-200.

Gabel NE (1949) A comparative racial study of the Papago. Albuquerque: University of New Mexico Publications in Anthropology. 96 p.

Ganguly P (1976) Physical Anthropology of the Nicobarese. New Delhi: Anthropological Survey of India, Government of India. 178 p.

Gates RT (1961) Todas and Kotas of the Nilgiri Hills. Mankind Quart 2: 98.

Gomila J (1971) Les Bedik (Senegal oreintal): Barrieres Culturelles et Heterogeneite Biologique. Montreal: Les Presses de 1’Universite de Montreal. 260 p.

Gould BA (1869) Investigations in the Military and Anthropological Investigations of American Soldiers. New York: Hurd and Houghton. 655 p.

Gould HN (1946) Anthropometry of the Chol Indians of Chiapas, Mexico. New Orleans: Middle American Research Institute. 32 p.

Hanna JM, Baker, PT (1974) Comparative heat tolerance of Shipibo Indians and Peruvian Mestizos. Hum Biol 46: 69-80.

Harper AB, Laughlin WS, Mazess RB (1984) Bone mineral content in St. Lawrence Island Eskimos. Hum Biol 56: 63-78.

Harvey RG (1974) An anthropometric survey of growth and physique of the populations of Karkar Island and Lufa subdistrict, New Guinea. Philos T Roy Soc B 268: 272-292.

Hastuti J (2009) Body composition in Javanese adults: some anthropometric dimensions related to body fat. Berkala Ilmu Kedokteran 41: 63-73.

Hiernaux J (1956) Analyse de la variation des caractères physiques humains en une région de l'Afrique Centrale: Ruanda-Urundi et Kivu. Ann Mus Congo Belge 3. 131 p.

Hiernaux J (1972) The analysis of multivariate biological distances between human populations: principles and applications to sub-Saharan Africa. In: Weiner JS, Huizinga J, editors. The Assessment of Population Affinities in Man. Oxford: Clarendon Press. 96-114.

Hiernaux J, Boedhi Hartono D (1980) Physical measurements of the adult Hadza of Tanzania. Ann Hum Biol 7: 339-346.

Holloway RL (1980) Within-species brain-body weight variability: A reexamination of the Danish data and other primate species. Am J Phys Anthropol 53: 109-121.

Howells WW (1937) Anthropometry of the natives of Arnhem Land and the Australian race problem: analysis and discussion. In: Papers of the Peabody Museum of American Archaeology and Ethnology, volume XVII, No. 1. Cambridge: Peabody Museum. 97 p.

Hurlich MG, Steegmann AT (1979) Hand immersion in cold water at 5°C in sub-arctic Algonkian Indian males from two villages: a European admixture effect? Hum Biol 51: 255-278.

Hurtado A (1928) Estudios de metabolismo basico en el Peru (Doctoral Dissertation). Lima: Universidad de San Marcos.

Katzmarzyk PT, Malina RM (1999) Body size and physique among Canadians of First Nation and European ancestry. Am J Phys Anthropol 108: 161-172.

Kirchengast S, Winkler E (1995) Differential reproductive success and body dimensions in Kavango males from urban and rural Areas in northern Namibia. Hum Biol 67: 291-309.

Kozlov A, Vershubsky G, Kozlova M (2007) Indigenous people of northern Russia: anthropology and health. Int J Circumpol Heal (Suppl.) 5-184.

Kumar S (2000) Encyclopedia of South-Asian tribes. New Delhi: Anmol Publications. 3442 p.

Leche SM (1936) Dermatoglyphics and functional lateral dominance in Mexican Indians. III. Zapotecas and Mixtecas. Anthropometry of the Zapotecas and Mixtecas. In: Cummins H, Lane MS, Leche SM, Millar R, Steggerda ID, Steggerda M, editors. Middle American Research Series 7: 229-239.

Lee M, Birkbeck JA (1977) Anthropometric measurements and physical examinations of Indian populations from British Columbia and the Yukon Territories, Canada. Hum Biol 49: 581–591.

Leonard WR, Katzmarzyk PT, Comuzzie AG, Crawford MH, Sukernik RI (1994) Growth and nutritional status of the Evenki reindeer herders of Siberia. Am J Hum Biol 6: 339-350.

Littlewood R (1972) Physical anthropology of the eastern highlands of New Guinea. Seattle: University of Washington Press. 224 p.

Macho G, Freedman L (1987) A Reanalysis of the Andrew A. Abbie Morphometric Data on Australian Aborigines. Occasional Paper on Human Biology 4, pp. 29-57. Canberra: Australian Institute of Aboriginal Studies.

Man EH, Rivers AP (1882) On the Andamanese and Nicobarese objects presented to Maj.-Gen. Pitt Rivers, F.R.S. J R Anthropol Inst G 11:268-294.

Milton K (1983) Morphometric features as tribal predictors in North-Western Amazonia. Ann Hum Biol 10: 435-440.

Neel JV, Salzano FM, Junqueira PC, Keiter F, Maybury-Lewis D (1964) Studies on the Xavante Indians of the Brazilian Mato Grosso. Ann J Hum Genet 16: 52-140.

Oyenhart EE, Techenski MF, Orden AB (2003) Nutritional status in two Mbyá-Guaraní communities from Misiones (Argentina). Homo 54: 170-179.

Patton JQ (1996) Thoughtful Warriors: Status, Warriorship, and Alliance in the Ecuadorian Amazon. (Doctoral dissertation) University of California, Santa Barbara.

Picón-Reátegui E, Buskirk ER, Doi K, Kuroshima A, Hiroshige T (1979) Anthropometric characteristics and body composition of Ainu and other Japanese: Comparison with other racial groups. Am J Phys Anthropol 50: 393-400.

Pi-Suner BJ (1933) Studies in racial metabolism: basal metabolism of the Araucanian Mapuches. Am J Physiol 105: 383-388.

Portman MV (1896) Notes on the Andamanese. J R Anthropol Inst G 25: 361-371.

Prokopec M (1977) An anthropometric study of the Rembarranga: comparison with other populations. J Hum Evo 6: 371-391.

Roberts DF (1953) Body weight, race and climate. Am J Phys Anthropol 11: 533-558.

Roux P (1905) Contribution à l'étude anthropologique de l'Annamite Tonkinois. B Mem Soc Anthro Par 6: 321-350.

Ruff CB, Niskanen M, Junno J, Jamison P (2005) Body mass prediction from stature and bi-iliac breadth in two high latitude populations, with application to earlier higher latitude humans. J Hum Evo 48: 381-392.

Sandstead HR, McGanity WJ, Smith HH, McKinley P, Timeche L, Darby WJ (1956) A Study of the Dietary Background and Nutriture of the Navajo Indian: III. Physical Findings. J Nutr 60 (Suppl. 2): 35-62.

Schlaginhaufen O (1964) Anthropology of New Ireland (Neumenklenburg) in the Melanesian South Sea. I. Observations on living natives. Arch Julius Klaus Stift Vererbungsforsch Sozialanthropol Rassenhyg 39: 1-237.

Shapiro HL (1939) Migration and Environment: A Study of the Physical-Characteristics of the Japanese Immigrants to Hawaii and the Effects of Environment on their Descendants. London: Oxford University Press. 594 p.

Singh K, Bhasin MK, Singh IP (2008) Age Changes in Biological Variables among High Altitude Bodh Males of Lahaul Tehsil, Lahaul-Spiti District, Himachal Pradesh, India. Anthropol 10: 193-202.

Sloan AW, Masali M (1978) Anthropometry of Sherpa Men. Ann Hum Biol 5: 453-458.

Slome C, Gamel B, Abranson H, Scotch N (1960) Weight, height and skinfold thickness of Zulu adults in Durban, 1958. S Afr Med J 34:505–509.

Sporcq J (1975) The Bira of the savanna and the Bira of the rain forest. A comparative study of two populations of the Democratic Republic of the Congo. J Hum Evo 4: 505-516.

Steggerda M (1932) Anthropometry of Adult Maya Indians: A study of their physical and physiological characteristics. Washington: Carnegie Institution of Washington, Publication No. 434. 113 p.

Stinson, S. (1989) Physical growth of Ecuadorian Chachi Amerindians. Am J Hum Biol 1: 697-707.

Walker R, Hill K (2003) Modeling growth and senescence in physical performance among the Ache of Eastern Paraguay. Am J Hum Biol 15: 196-208.

Winkler EM, Kirchengast S (1994) Body dimensions and differential fertility in !Kung San males from Namibia. Am J Hum Biol 6: 203-213.

Wyndham CH, Ward JS, Strydom NB, Morrison JF, Williams CG, Bredell GAG, Peter J, Von Rahden MJE, Holdsworth LD, Van Graan CH, Van Rensburg AJ, Munro A (1964a) Physiological reactions of Caucasian and Bantu males in acute exposure to cold. J Appl Physiol 19: 583-592.

Wyndham CH, Morrison JF, Ward JS, Bredell GAG, Von Rahden MJE, Holdsworth LD, Wenzel HG, Munro A (1964b) Physiological reactions to cold of Bushmen, Bantu, and Caucasian males. J Appl Physiol 19: 868-876.
